# Development of Fluoride-Ion Primary Batteries: The Electrochemical Defluorination of CF_*x*_

**DOI:** 10.1021/acs.jpcc.4c03412

**Published:** 2024-08-15

**Authors:** Loleth E. Robinson, Jonah Wang, Harrison Asare, Jessica L. Andrews, Balram Tripathi, Ram Katiyar, Brent C. Melot, Robert J. Messinger, Simon C. Jones, William C. West

**Affiliations:** † Department of Chemical Engineering, The City College of New York, CUNY, New York, New York 10031, United States; ‡ Department of Chemistry and Biochemistry, The City College of New York, CUNY, New York, New York 10031, United States; § Ph.D. Program in Chemistry, The Graduate Center of the City University of New York, New York, New York 10016, United States; ∥ Department of Chemistry, University of Southern California, Los Angeles, California 90089, United States; ⊥ Department of Chemical Engineering and Materials Science, University of Southern California, Los Angeles, California 90089, United States; # Department of Physics and Institute for Functional Nanomaterials, University of Puerto Rico, San Juan 00925-2537, Puerto Rico; ∇ Flion Energy Inc., Pasadena, California 91107, United States; ○ Jet Propulsion Laboratory, California Institute of Technology, Pasadena, California 91109, United States

## Abstract

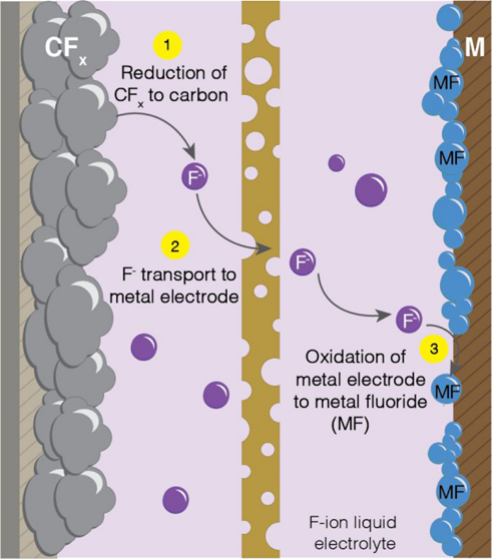

The lithium–carbon monofluoride (Li-CF_*x*_) couple has the highest specific energy of any practical battery chemistry. However, the large polarization associated with the CF_*x*_ electrode (>1.5 V loss) limits it from achieving its full discharge energy, motivating the search for new CF_*x*_ reaction mechanisms with reduced overpotential. Here, using a liquid fluoride (F)-ion conducting electrolyte at room temperature, we demonstrate for the first time the electrochemical defluorination of CF_*x*_ cathodes, where metal fluorides form at a metal anode instead of the CF_*x*_ cathode. F-ion primary cells were developed by pairing CF_*x*_ cathodes with either lead (Pb) or tin (Sn) metal anodes, which achieved specific capacities of over 700 mAh g^–1^ and over 400 mAh g^–1^, respectively. Solid-state ^19^F and ^119^Sn{^19^F} nuclear magnetic resonance (NMR), X-ray diffraction (XRD), Raman, inductively coupled plasma (ICP), and X-ray fluorescence (XRF) measurements establish that upon discharge, the CF_*x*_ cathode defluorinates while Pb forms PbF_2_ and Sn forms both SnF_4_ and SnF_2_. Technological development of F-ion metal-CF_*x*_ cells based on this concept represents a promising avenue for realizing primary batteries with high specific energy.

## Introduction

The lithium–carbon monofluoride (Li-CF_*x*_) primary (nonrechargeable) battery cell is employed across a broad spectrum of commercial uses, from implantable medical devices to marine, military, medical, and space applications.^1−4^ This battery chemistry offers the highest specific energy (∼2180 Wh kg^–1^, based on a theoretical discharge potential of 4.57 V)^1^ and specific capacity (865 mAh g^–1^, based on CF_*x*_ cathode when *x* = 1) among all lithium nonrechargeable chemistries commercially available.^2^ In many cases, the limited capacity of power sources is the key performance bottleneck for various technologies that underpin modern technological advancements; therefore, greater energy is always desirable. For example, the Li-CF_*x*_ chemistry is of significant interest for future spacecraft applications, especially where a photovoltaic or radioisotope power is either unavailable or impractical.^5,6^ As applications become more complex and more energy-demanding, achieving higher practical energy output for longer use times is critical, highlighting the need to identify new cell chemistries.

The electrochemical conversion mechanism for the conventional Li-CF_*x*_ cell discharge process is (1) the anodic oxidation of the lithium metal anode, Li → Li^+^ + e^–^, (2) the transport of lithium ions through the electrolyte, and (3) the cathodic reduction of the CF_*x*_ to form carbon (C) and lithium fluoride (LiF) with the discharge products remaining at the cathode, CF_*x*_ + *x*Li → (LiF)_*x*_ + C. The overall spontaneous electrochemical conversion reaction is CF_*x*_ + *x*Li^+^ + *x*e^–^ → (LiF)_*x*_ + C.

Although the theoretical discharge voltage of the Li-CF_*x*_ cell is >4 V, the practical discharge voltage (∼2.5 V), even at low current densities, is significantly lower than the theoretical value. This ca. 2 V polarization loss represents a massive penalty in specific energy relative to the theoretical value. For example, a Li-CF_*x*_ D-cell discharged at 2.5 V offers a specific energy of 700 Wh kg^–1^. If the same cell was discharged at 4.5 V, then the specific energy achieved is closer to 1260 Wh kg^–1^, a 560 Wh kg^–1^ (or 80%) practical gain. Most importantly, the higher energy output provided by a 4.5 V discharge voltage design could replace other primary cell chemistries such as lithium-thionyl chloride (Li-SOCl_2_) without the need to change the power electronics design. The low practical discharge voltage of Li-CF_*x*_ has been ascribed to several mechanisms.^7−12^ Whittingham^9^ proposed a two-phase reaction mechanism, where the discharge product is a nonstoichiometric ternary intercalation compound (CLi_*x*_F) that later decomposes into carbon and LiF. Nonstoichiometric solvent-mediated ternary compounds have also been proposed (CFLi_*x*_:S_*y*_), where co-intercalated Li^+^ and solvent molecules later decompose to the final discharge products.^10,12^ Recently, Leung et al.^8^ proposed a solvent-mediated edge propagation mechanism and demonstrated a correlation between the solvent-based lithium intercalation complex and the CF_*x*_ operating voltage window using density functional theory methods.

We hypothesize that by fundamentally changing the discharge process of the Li-CF_*x*_ cell chemistry, it may be possible to access a greater discharge potential. One possible route to do so is to change the discharge process from lithiating the CF_*x*_ cathode to defluorinating the CF_*x*_ cathode and fluorinating the anode. Clearly, the discharge mechanism and location of the discharge product (metal fluorides) are entirely different in this cell relative to conventional metal anode −CF_*x*_ cells, which raises the possibility of improvement to a higher practical discharge voltage. The reversible fluorination of CuF_2_ and BiF_3_ has been demonstrated previously; however, applications are limited since high temperatures (∼150 °C) are necessary to cycle the battery at practical rates using a solid F-ion conductor.^13,14^ Recent reports describe the development of a room temperature fluoride-ion conducting electrolyte.^15,16^ However, a compatible metal anode is necessary, given the strong reactivity between the lithium metal and the F-ion electrolyte.

In this work, using a liquid F-ion conducting electrolyte, we demonstrate the electrochemical defluorination of CF_*x*_ electrodes at room temperature versus either lead Pb or Sn metal anodes. Solid-state ^19^F and ^119^Sn{^19^F} nuclear magnetic resonance (NMR), X-ray diffraction (XRD), inductively coupled plasma (ICP), Raman, and X-ray fluorescence (XRF) measurements evidence the defluorination of the CF_*x*_ electrode upon discharge and concurrent formation of PbF_2_ at the Pb anode or SnF_4_ and SnF_2_ at the Sn anode. XRD, ICP, and XRF revealed that only modest transport of either Pb or Sn between electrodes occurred; however, cation transport is reduced substantially when using Sn. To the best of our knowledge, this is the first report of the electrochemical defluorination of a CF_*x*_ electrode, which is reduced to carbon, with the concomitant fluorination of a metal anode as the conjugate electrochemical reaction.

## Methods

### Electrode and Cell Preparation

The CF_*x*_ cathodes were prepared in-house by mixing 92 wt % CF_*x*_ powder (ARC, *x* ≈ 1, theoretical capacity of 865 mAh g^–1^), 5 wt % carbon black (Super P, Alfa Aesar), and 3 wt % poly(vinylidene fluoride) (PVDF; Aldrich) dissolved in *N*-methyl-2-pyrrolidone (NMP; Aldrich) to prepare a slurry. Note that *x* ≈ 1 in CF_*x*_ represents the average composition of the bulk active material; however, local differences in fluorination are expected, giving rise to compositional heterogeneity within the CF_*x*_ structure. Self-standing Pb metal electrodes were prepared using a composition of 80 wt % Pb metal powder (CERAC Inc., 200 mesh), 10 wt % carbon black, and 10 wt % poly(tetrafluoroethylene) (PTFE; Aldrich). The Pb slurry was prepared in a mortar and pestle using acetone as a solvent and later calendared using a manual laboratory roller to form a self-standing electrode. The Sn metal electrodes were also prepared using a slurry composed of 80 wt % Sn metal powder (CERAC Inc.), 10 wt % carbon black, and 10 wt % PVDF dissolved in *N*-methylpyrrolidone (NMP). Prior to casting, the Sn electrode powdered components were ball milled (SPEX 8000) for 2 h. The CF_*x*_ and Sn slurries were cast on a 0.127 mm thick stainless steel foil (Shop-Aid, Inc.) precoated with a layer of carbon ink (EB-012 Henkel Corp). All electrodes were dried overnight at 100 °C under vacuum prior to cell assembly.

The composite CF_*x*_ cathode, Sn anode, and Pb anode mass loadings were 5.93, 23, and 248 mg cm^–2^, respectively. Stainless steel CR2032 coin cell cases were stripped from their interior aluminum coating to prevent undesired reactions with the electrolyte solvent. To remove the aluminum coating from the coin cell casings, they were placed in a 1 M solution of NaOH (J.T. Baker) for 20 min; then, they were rinsed with deionized water and dried overnight. Coin cells were prepared using dried CF_*x*_ and Sn metal electrodes with 16 mm diameter, Pb metal electrodes with 13 mm diameter, separators (Celgard 2325) with 17 mm diameter, and 100 μL of electrolyte consisting of 0.75 M *N*,*N*,*N*-trimethyl-*N*-neopentylammonium fluoride (Np_1_F) dissolved in bis(2,2,2-trifluoroethyl) ether (BTFE; Aldrich), under an argon atmosphere (H_2_O and O_2_ < 1 ppm). The Np_1_F fluoride salt was synthesized as described in detail by Davis et al.^15^ Following manual crimp sealing, all coin cells were galvanostatically discharged using an Arbin LBT battery tester at 10 μA discharge current to a cutoff voltage of 0 V. All metal-CF_*x*_ cells were designed with anode specific capacities far in excess of the cathode specific capacities; thus, they are cathode-limited in capacity. All specific capacities are reported per mass of CF_*x*_ (865 mAh g^–1^, based on the CF_*x*_ cathode when *x* ≈ 1). Calculations of the specific capacity are detailed in Text S1, Supporting Information.

### X-ray Diffraction

Powder XRD measurements were carried out using an Aeris PANalytical X-Pert system with a diffractometer run in the θ – 2θ geometry, with a Cu anode (λ = 1.541 A) at an accelerating voltage of 40 kV and a tube current of 15 mA, standard phase ID measurement profile measured from 10 to 100° 2θ using a divergence slit = 0.125°, Soller slit = 0.02 rad, with step size = 0.01°.

The resulting XRD patterns for both the pristine and discharged Pb and Sn electrodes were refined against published structures using the Rietveld method as implemented in the TOPAS-Academic suite,^17^ with the resulting structural parameters given in Tables S1–S4.

For XRD sample preparation, CF_*x*_, Sn, and Pb electrodes were harvested from discharged coin cells inside a dry room with H_2_O content <5 ppm. Ethanol (Fischer Scientific) was used as a rinsing solvent to remove residual organic electrolyte from the harvested electrodes prior to measurements. Pristine CF_*x*_ and Sn electrodes were used as prepared. The interlayer distance extracted from the diffraction pattern of the pristine CF_*x*_ electrode is 0.7 nm.

### Scanning Electron Microscopy (SEM)

SEM was performed by using a Zeiss Supra 55 field emission scanning electron microscope under high vacuum. The pristine CF_*x*_ electrode was gold plated prior to imaging to prevent surface charging.

### Inductively Coupled Plasma-Optical Emission Spectroscopy (ICP-OES)

Inductively coupled plasma-optical emission spectroscopy (ICP-OES) was carried out using a PerkinElmer Optima 7300 DV ICP operating with an argon plasma flow of 15 and 0.2 L min^–1^ auxiliary flow. Nebulizer flow was 0.55 L min^–1^ with a peristaltic pump sample introduction flow rate of 1.5 mL min^–1^. Radio frequency (rf) power was 1450 W, and the instrument optics was set for an axial view of the plasma. The ICP was calibrated using a top-level standard containing Sn and Pb, present at 1.0 ppm (1.0 μg mL^–1^), in 2% HNO_3_ (prepared from Ultrex ultrapure nitric acid and deionized water). For standards and samples, triplicate runs were performed, and the results were averaged. The electrodes were placed into ICP vials containing ∼5 g of ASTM type I water and allowed to stand overnight. Samples were analyzed as-is, along with an ASTM type I water sample as a blank.

### Raman Spectroscopy

Raman spectra were measured using the excitation wavelength of 514.5 nm of an argon ion laser at a power of 1 mW.

### X-ray Fluorescence Spectroscopy

XRF measurements were carried out using a Horiba model XGT-9000 X-ray fluorescence microscope (μXRF), using the following settings: live time: 100 s; processing time: P2; XGT diameter: 50 μm; X-ray tube voltage: 30 kV; current: automatic. Samples were scanned at two different locations with a 1.2 mm beam size and averaged to increase the area response.

### Solid-State Nuclear Magnetic Resonance Spectroscopy

Solid-state NMR experiments were acquired on a Bruker Avance III HD 600 NMR spectrometer with a 14.1 T narrow-bore superconducting magnet operating at 564.69 and 223.79 MHz for ^19^F and ^119^Sn nuclei, respectively. A Phoenix NMR 1.6 mm HXY magic-angle-spinning (MAS) probehead was used, where all measurements were conducted with MAS rates of 40 kHz unless specified otherwise. Air was pumped through the probehead at 293.2 K and 600 L h^–1^ to mitigate sample heating due to MAS. ^19^F chemical shifts were referenced to CFCl_3_ at 0 ppm using pristine LiF (Sigma-Aldrich, ≥99.99%) at −204 ppm as a secondary chemical shift reference. ^7^Li shifts were referenced to 1 M aqueous LiNO_3_ at 0 ppm using a pristine LiF shift at −1 ppm as a secondary chemical shift reference. ^119^Sn chemical shifts were referenced indirectly by scaling the ^119^Sn frequency with respect to ^7^Li nuclei by using the ratio of their gyromagnetic ratios, γ_119_Sn__/γ_7_Li__ (0.965).

Solid-state ^19^F experiments used radio frequency (rf) field strengths of 147 kHz (π/2 of 1.7 μs) for all broad-band rf pulses. Solid-state ^19^F spin-echo NMR experiments were acquired to eliminate the ^19^F probe and rotor background using a half-echo delay of six rotor periods (150 μs). Recycle delays (25 s) were calibrated such that all ^19^F nuclear spins relaxed to thermal equilibrium (>5 × *T*_1_, the longitudinal relaxation time) between scans. For the CF_*x*_ electrode, the relative populations of the ^19^F signals of the solid-state ^19^F spin-echo NMR experiments can be quantitatively analyzed because the total echo delay of 300 μs is smaller than the transverse ^19^F longitudinal (*T*_2_) relaxation times of the different ^19^F signals (ranged from 1.5 to 6.7 ms). Two-dimensional (2D) ^19^F{^19^F} finite pulse radiofrequency-driven recoupling (fp-RFDR) NMR experiments were acquired to probe the molecular-scale proximities of ^19^F moieties using mixing times of 1.6 ms. The fp-RFDR π pulses were phase cycled according to the (XY-8)4^1^ super cycle, where the ratio of the π pulse duration (1.7 μs) to the rotor period (25 μs) was 0.068.

To enhance ^119^Sn signal sensitivity, solid-state ^119^Sn{^19^F} cross-polarization magic-angle-spinning (CP-MAS) experiments were used to characterize ^119^Sn nuclei. ^19^F and ^119^Sn rf field strengths of 100 and 60 kHz were used, corresponding to the zero-quantum *n* = +1 Hartmann–Hahn matching condition. ^119^Sn{^19^F} CP-MAS contact times of 4 ms were used for all experiments. The solid-state 2D ^119^Sn{^19^F} heteronuclear correlation (HETCOR) NMR experiments are 2D analogues of the one-dimensional (1D) ^119^Sn{^19^F} CP-MAS NMR conditions, enhancing the spectral resolution. STATES-TPPI quadrature detection was used in the indirect dimension for all 2D experiments.

For NMR sample preparation, CF_*x*_ and Sn electrodes were harvested from discharged coin cells inside an argon-filled glovebox (H_2_O < 1.0 ppm and O_2_ < 1 ppm). Coin cells were decrimped using an MTI electric (de)crimping machine (model MSk-160E), where the decrimping pressure was set to 2016 psi. The active material was stripped from the stainless steel current collector, crushed using a mortar and pestle, and packed in a 1.6 mm diameter zirconia rotor inside the glovebox. Commercial SnF_2_ (Thermo Fisher Scientific, 97.5%) and SnF_4_ (Thermo Fisher Scientific, 99%) were used as purchased and packed in the glovebox. Solid-state NMR spectra were deconvoluted using DMFit.^18^

## Results and Discussion

### Electrochemical Performance of F-Ion Sn-CF_*x*_ and Pb-CF_*x*_ Primary Cells

**Figure 1:**
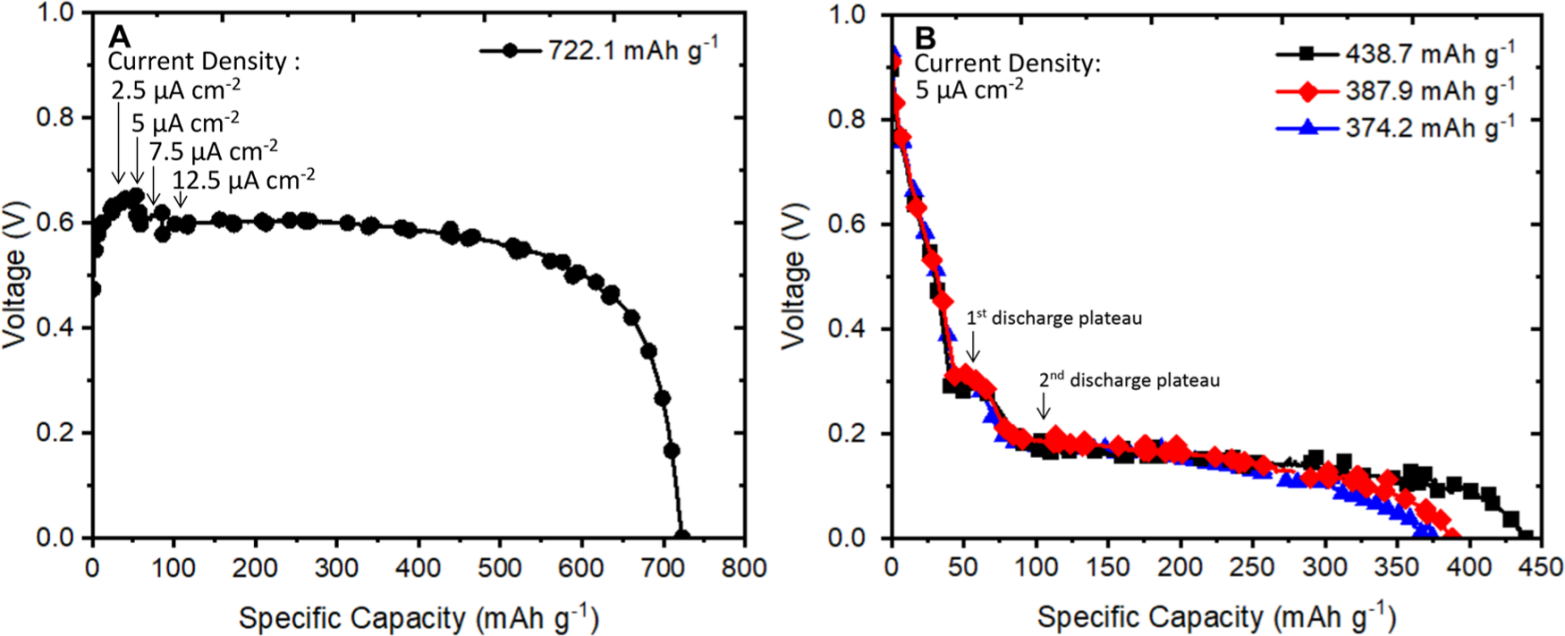
(A) Galvanostatic discharge of a F-ion Pb-CF_*x*_ primary cell at the current densities noted. (B) Galvanostatic discharge of F-ion Sn-CF_*x*_ primary cells discharged at a rate of 5 μA cm^–2^.

To electrochemically defluorinate the CF_*x*_ cathode, the C–F bonds in the CF_*x*_ active material must be broken, followed by the F-ions migrating to the anode through a fluoride-ion conducting electrolyte, where they react with the reduced metal ion to form a fluorinated metal discharge product at the anode. The liquid fluoride-ion conducting electrolyte, 0.75 M Np_1_F dissolved in BTFE, offers a wide electrolyte operating voltage window (4.1 V) and high ionic conductivity (10^–3^–10^–2^ S cm^–1^) that allows for compatibility with a variety of metals.^15^ We tested the electrochemical discharge performance of the CF_*x*_ electrode against different metal counter electrodes. A CF_*x*_ cell against a Pb metal counter electrode was prepared and discharged to 0 V (Figure 1A). The Pb-CF_*x*_ cell was tested by applying a low initial current of 2.5 μA cm^–2^, and the current was gradually increased to determine the bounds of the practical current necessary to effectively discharge the cell. Once the current density increased to 12.5 μA cm^–2^, the characteristic flat discharge plateau of CF_*x*_ was observed at ca. 0.6 V and achieved a specific capacity of 722.1 mAh g^–1^.

As shown above, ICP measurements of the harvested CF_*x*_ electrode from the Pb-CF_*x*_ cell after discharge revealed that the PbF_2_ discharge product was present at both the anode and cathode, indicating that mass transport of a Pb species is occurring. While the precise lead species is unknown, if the PbF_2_ was formed due to Pb^2+^ transport and reaction according to *x*Pb^2+^ + 2CF_*x*_ + 2*x*e^–^ → *x*PbF_2_ + 2C at the CF_*x*_ electrode, we estimate that 5.4% of the discharge capacity was due to Pb^2+^ ion transport and electrochemical PbF_2_ formation, with the balance of discharge capacity (94.6%) proceeding by the electrochemical defluorination of CF_*x*_ and subsequent F-ion transport.

Importantly, the voltage of the F-ion Pb-CF_*x*_ cell is strongly suggestive that a much higher operating voltage of the CF_*x*_ cathode can be obtained in this novel cell design if a Li anode were used. For example, the discharge plateau of the F-ion Pb-CF_*x*_ cell is approximately 0.6 V. Comparing the standard half-cell potentials of Pb (−0.13 V vs standard hydrogen electrode (SHE) for Pb → Pb^2+^ + 2e^–^) and Li (−3.01 V vs SHE for Li → Li^+^ + e^–^),^19^ replacing the Pb anode with a Li anode would result in a ca. 3.5 V cell vs Li/Li^+^ (activities of the reactants and products associated with the different half-cell reactions would result in an additional minor correction). This calculation indicates that the CF_*x*_ discharge plateau would be closer to the theoretical discharge potential of CF_*x*_ (e.g., compared to the 2.5–2.7 V typically observed for Li-CF_*x*_). Thus, replacing Pb with Li could also allow for a much greater discharge voltage.

Sn metal was chosen in an attempt to find a compatible anode material with reduced cation transport. F-ion Sn-CF_*x*_ cells were fabricated, using a 3-fold increase in the Sn electrodes mass loading to ensure that the CF_*x*_ is the capacity-limiting electrode. The electrochemical performance of several Sn-CF_*x*_ cells was observed by discharging cells under a constant current density of 5 μA cm^–2^, from the open-circuit voltage (OCV) of ∼1.0 to 0 V (Figure 1B). Specific capacities ranging from 374.2 to 438.7 mAh g^–1^ were observed and presumed to be dependent on the extent of defluorination of the CF_*x*_ electrode. A small initial voltage plateau was observed at 0.27 V, followed by the main voltage plateau at approximately 0.15 V. The two discharge plateaus correspond to the formation of two fluorinated discharge products, SnF_2_ and SnF_4_, as shown below. Notably, the Sn-CF_*x*_ cell approached roughly ∼50% of the theoretical capacity of CF_*x*_ (865 mAh g^–1^ when *x* = 1), without optimization, at room temperature.

As shown below, solid-state NMR measurements of the harvested Sn electrode from the Sn-CF_*x*_ cell revealed SnF_4_ present at the CF_*x*_ electrode, which is consistent with XRF measurements. This result indicates that there is mass transport of a Sn species from the anode to the CF_*x*_ cathode. While the precise tin species is unknown, if the SnF_2_ formed due to Sn^2+^ transport to the CF_*x*_ cathode and reaction according to *x*Sn^2+^ + 2CF_*x*_ + 2*x*e^–^ → *x*SnF_2_ + 2C. In principle, Sn could further electrochemically oxidize (Sn^2+^ →Sn^4+^ + 2e^–^) at the tin metal anode, with subsequent electrochemical reduction at the CF_*x*_ cathode to *x*Sn^4+^ + 4CF_*x*_ + 4*x*e^–^ → *x*SnF_4_ + 4C. We estimate that 3.1% of the discharge capacity was achieved by Sn^2+^ transport, a >40% relative decrease from the Pb^2+^ case. Importantly, we estimate that electrochemically defluorinating CF_*x*_ reduces the overall polarization loss by approximately 1.1 and 0.6 V when using Pb and Sn (e.g., compared to approximately 2 V polarization loss observed when lithiating CF_*x*_), respectively.

### Electrode Crystal Structures and Morphologies

**Figure 2:**
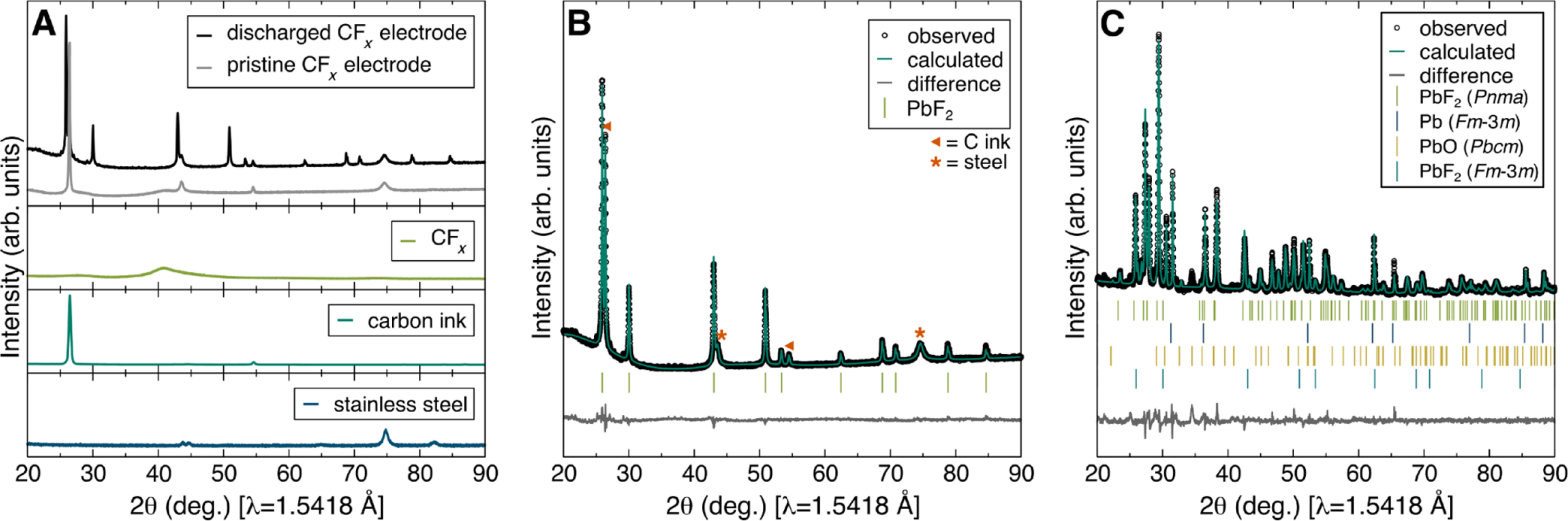
XRD pattern of a (A) discharged CF_*x*_ electrode from an F-ion Pb-CF_*x*_ cell (black), pristine CF_*x*_ electrode (gray), CF_*x*_ powder (light green), carbon ink (dark green), and stainless steel foil (blue). (B) Rietveld refinement of a CF_*x*_ electrode from a discharged F-ion Pb-CF_*x*_ cell. (C) Rietveld refinement of a discharged Pb electrode from a F-ion Pb-CF_*x*_ cell.

Powder XRD measurements were acquired on a pristine CF_*x*_ electrode and discharged CF_*x*_ and Pb electrodes to characterize the structural changes after discharge (Figure 2). The pristine CF_*x*_ electrode (Figure 2A, gray) exhibits two reflections that correspond to the carbon ink precoating at ca. 2θ of 27 and 54°, as well as two reflections from the stainless steel current collector at ca. 2θ of 44 and 75°. The broad CF_*x*_ reflection at ca. 2θ of 42° is consistent with the disordered nature of the CF_*x*_ structure. The XRD pattern of the CF_*x*_ electrode after discharge against the Pb anode (Figure 2A, black) exhibits multiple new reflections between ca. 2θ of 40 and 79° corresponding to PbF_2_ in cubic space group *Fm*3̅*m* (no. 225). Rietveld refinements of this structure against the XRD data revealed the presence of cubic PbF_2_ in both the discharged CF_*x*_ and Pb electrodes, evidencing the transport of Pb ions to the CF_*x*_ cathode during discharge (Figure 2B,C). The multiphase Rietveld refinement of the discharged Pb anode also indicates the presence of orthorhombic PbF_2_ (*Pnma*, no. 62), cubic Pb (*Fd*3̅*m*, no. 225), and an orthorhombic PbO impurity (*Pbcm*, no. 57). The PbO impurity was likely introduced during the electrode casting process, where high surface area Pb metal powder was ground at ambient conditions.

**Figure 3:**
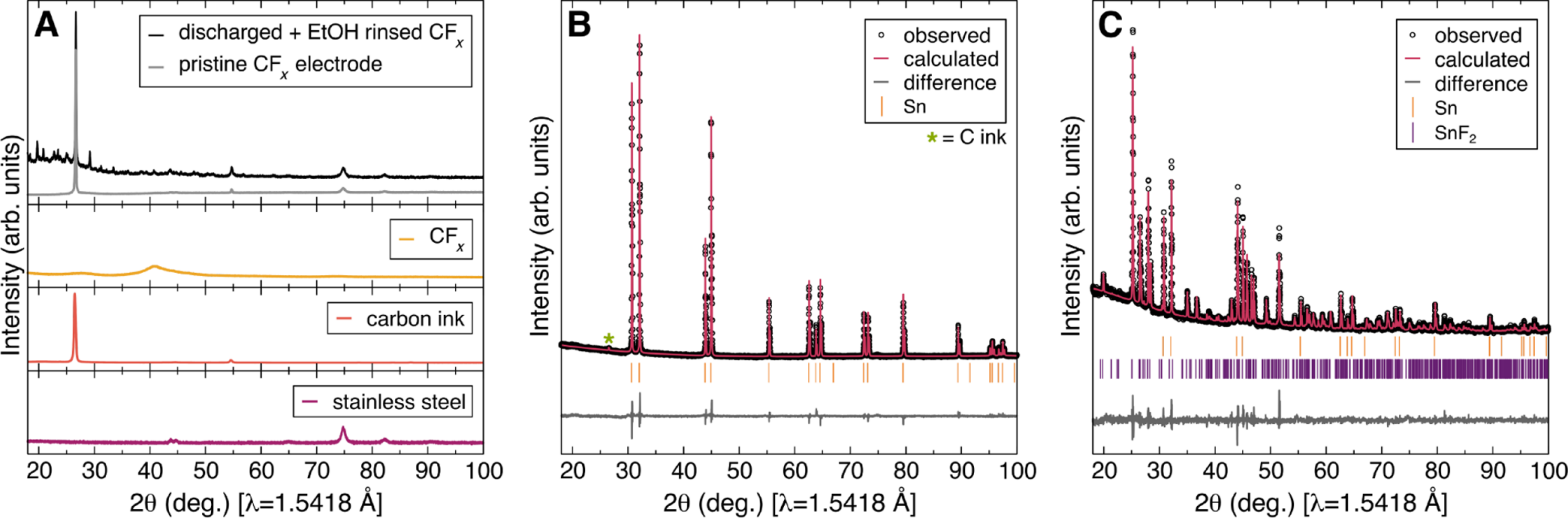
XRD pattern of (A) discharged CF_*x*_ electrode from a F-ion Sn-CF_*x*_ cell (black), pristine CF_*x*_ electrode (gray), CF_*x*_ powder (yellow), carbon ink (orange), and stainless steel foil (purple). Rietveld refinement of (B) pristine Sn electrode and (C) discharged Sn electrode from an F-ion Sn-CF_*x*_ cell.

XRD measurements were also collected on a CF_*x*_ cathode discharged against a Sn anode, as well as the corresponding discharged Sn electrode (Figure 3). The CF_*x*_ electrode (Figure 3A, black) reveals multiple low-angle reflections associated with the residual electrolyte (Figure S1). Critically, reflections associated with Sn, as observed in the pristine Sn electrode (Figure 3B), or a Sn–F binary phase were not detected in the XRD pattern of the discharged CF_*x*_ electrode, suggesting that Sn ion transport is minimal during discharge. The XRD data for the discharged Sn electrode (Figure 3C) exhibit reflections corresponding to both tetragonal Sn (*I*4_1_/*amd*, no. 141) and monoclinic SnF_2_ (*C*12/*c*1, no. 15).

**Figure 4:**
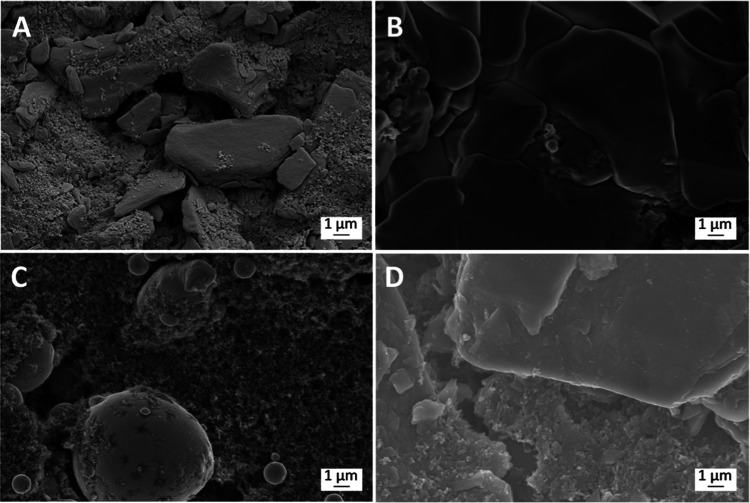
SEM images of (A) pristine and (B) discharged CF_*x*_ electrode and (C) pristine and (D) discharged Sn electrode. Both the CF_*x*_ and Sn discharged electrodes were harvested from an F-ion Sn-CF_*x*_ cell. The pristine CF_*x*_ electrode was gold plated prior to imaging to prevent surface charging.

Morphological changes in the CF_*x*_ and Sn electrodes were also visualized before and after discharge using SEM (Figure 4). The surface morphology of the CF_*x*_ electrode becomes considerably smoother upon discharge. Note that in Li-CF_*x*_ cells, insulating LiF is formed during electrochemical discharge; here, no additional discharge products are observed, consistent with the electrochemical defluorination mechanism proposed here. The composite Sn metal electrode exhibits a relatively inhomogeneous distribution of active material, a result of the difficulty in casting uniform Sn electrodes due to Sn metal being a dense active material.

### Elemental and Surface Analysis

ICP measurements were acquired on a CF_*x*_ and Pb electrode after discharge to quantify the Pb content in each electrode after discharge. Each electrode was placed into deionized water, where soluble Pb ions from the lead fluoride discharge products were hydrolyzed and quantified. The CF_*x*_ and Pb electrodes were found to contain hydrolyzed PbF_2_. As discussed above, we estimate that 5.4% of the discharge capacity was due to Pb^2+^ ion transport, with the balance of discharge capacity (94.6%) proceeding by F^–^ ion transport.

ICP acquired on a discharged Sn electrode reveals that the Sn fluoride discharge products contain 4.77 mg of Sn and 1.52 mg of fluorine. The estimated mass of fluorine removed from CF_*x*_ based on the specific capacity achieved is 1.58 mg. ICP was not acquired on the CF_*x*_ electrode; however, the good agreement between the hydrolyzed metallic fluoride content in the Sn anode and electrochemical measurements indicates that at least 96.2% of the discharge product was formed in the Sn anode.

XRF spectroscopy was used to determine and compare the elemental composition of a pristine CF_*x*_ electrode and a CF_*x*_ electrode discharged to 0 V. Results reveal a nondetectable weight percentage of Sn metal in the pristine CF_*x*_ cathode and 2.5 wt % once discharged. The fluorine content of the pristine CF_*x*_ electrode was 37.2 wt %. Once discharged, the fluorine content decreased to 6.2 wt %, indicating an 83.3 wt % decrease in fluorine content after discharge of the CF_*x*_ electrode. Given the composition of the electrode, the fluorine content solely from the PVDF binder can be estimated to be 1.69 wt %. After accounting for the binder, the estimated mass of fluorine removed from the sample based on electrochemical measurements is 3.10 mg. From the XRF analysis, the estimated fluorine content removed was 3.51 mg, a value in good agreement with our electrochemical results. Solid-state NMR measurements of the harvested Sn electrode from the Sn-CF_*x*_ cell revealed that SnF_4_ was present at the CF_*x*_ electrode. We estimate that 3% of the discharge capacity was achieved by Sn^2+^ transport. Estimates of the quantity of metal fluorides present on the CF_*x*_ electrodes through ICP, XRF, and solid-state NMR are further described in Text S2.

**Figure 5:**
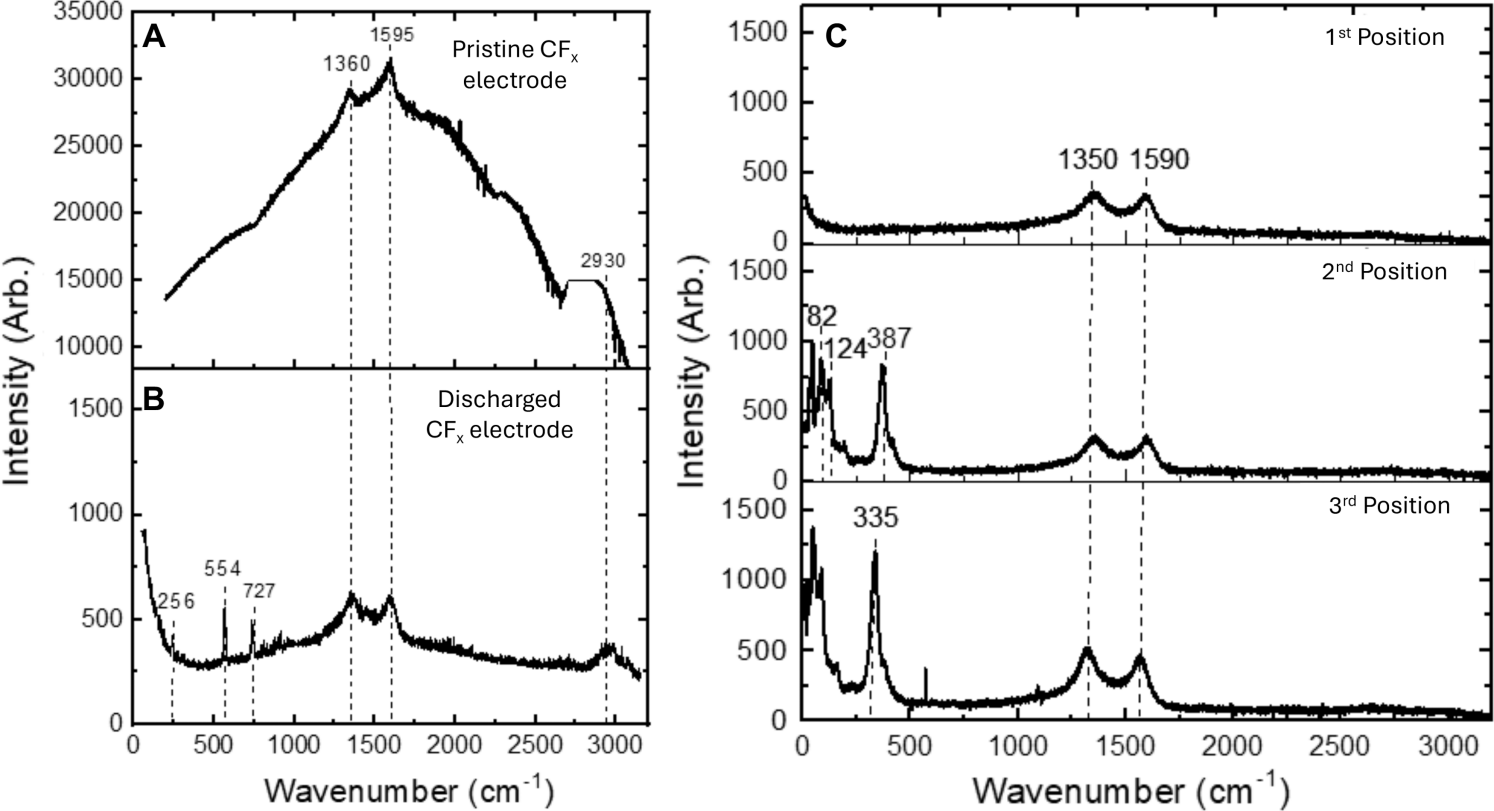
Raman scattering from (A) pristine CF_*x*_ electrode and (B) discharged CF_*x*_ electrode. Raman scattering from a (C) discharged Sn electrode was performed at three different positions. Both the CF_*x*_ and Sn discharged electrodes were harvested from an F-ion Sn-CF_*x*_ cell.

Raman scattering from pristine CF_*x*_ (Figure 5A) shows a strong D peak at 1360 cm^–1^, *G*′ peak at 1595 cm^–1^, and D + G peak at 2930 cm^–1^ with strong luminescence from the samples. Raman features of carbon and its derivatives have been extensively studied, and the D peak defines the presence of defects, e.g., lattice dislocation, vacancies, etc., for its activation. The D peak usually appears on the high-frequency shoulder of the G peak, and it activates upon a symmetry break in the carbon plane.

We also performed Raman scattering measurements on a harvested CF_*x*_ cathode discharged against a Sn counter electrode (Figure 5B). In the case of discharged CF_*x*_, the luminescence is considerably quenched, and D, G, and D + G peaks broaden and are clearly visible in co-occurrence with a significant increase of the D/G peak ratios in comparison to pristine CF_*x*_. The D peak arises only from clusters of sp^2^ sites, and the G peak arises from vibrations of all sp^2^ sites in both chain and ring configurations. However, some more peaks arise at 256, 554, and 727 cm^–1^, showing the changes in Raman spectra are most likely caused by the structural disordering in the carbon lattice, e.g., the transformation of sp^2^ carbon to sp^3^ hybridization because of the fluoride-ion adsorption. The Raman signature of CF_*x*_ after discharge exhibits a characteristic similar to that from highly disordered or nanostructured carbon-based materials.

We also investigated the Raman spectra of the discharged Sn electrode at three positions of the electrode surface (Figure 5C). We did not find any characteristic peak of SnO_2_ at any point. As shown in Raman spectra, we found SnF_2_ peaks at 387 cm^–1^ (A_1g_), 124 cm^–1^ (E_g_), and 82 cm^–1^ (B_1g_) due to crystallized SnF_2_ (Figure 5C, second position). At some other points, we have found a strong peak at 335 cm^–1^, which is believed to be due to the amorphous structure of SnF_2_, suggesting formations of nano-ordered regions (Figure 5C, third position). The Raman modes at lower frequencies are due to the amorphous nature of the material.

### Molecular-Level Compositions and Environments

Solid-state ^19^F spin-echo MAS and ^119^Sn{^19^F} CP-MAS NMR experiments were acquired on pristine and discharged Sn and CF_*x*_ electrodes to characterize the molecular-level environments of the fluorine and tin discharge products. To aid signal assignments, commercially purchased SnF_2_ and SnF_4_, as well as PVDF, were also characterized.

**Figure 6:**
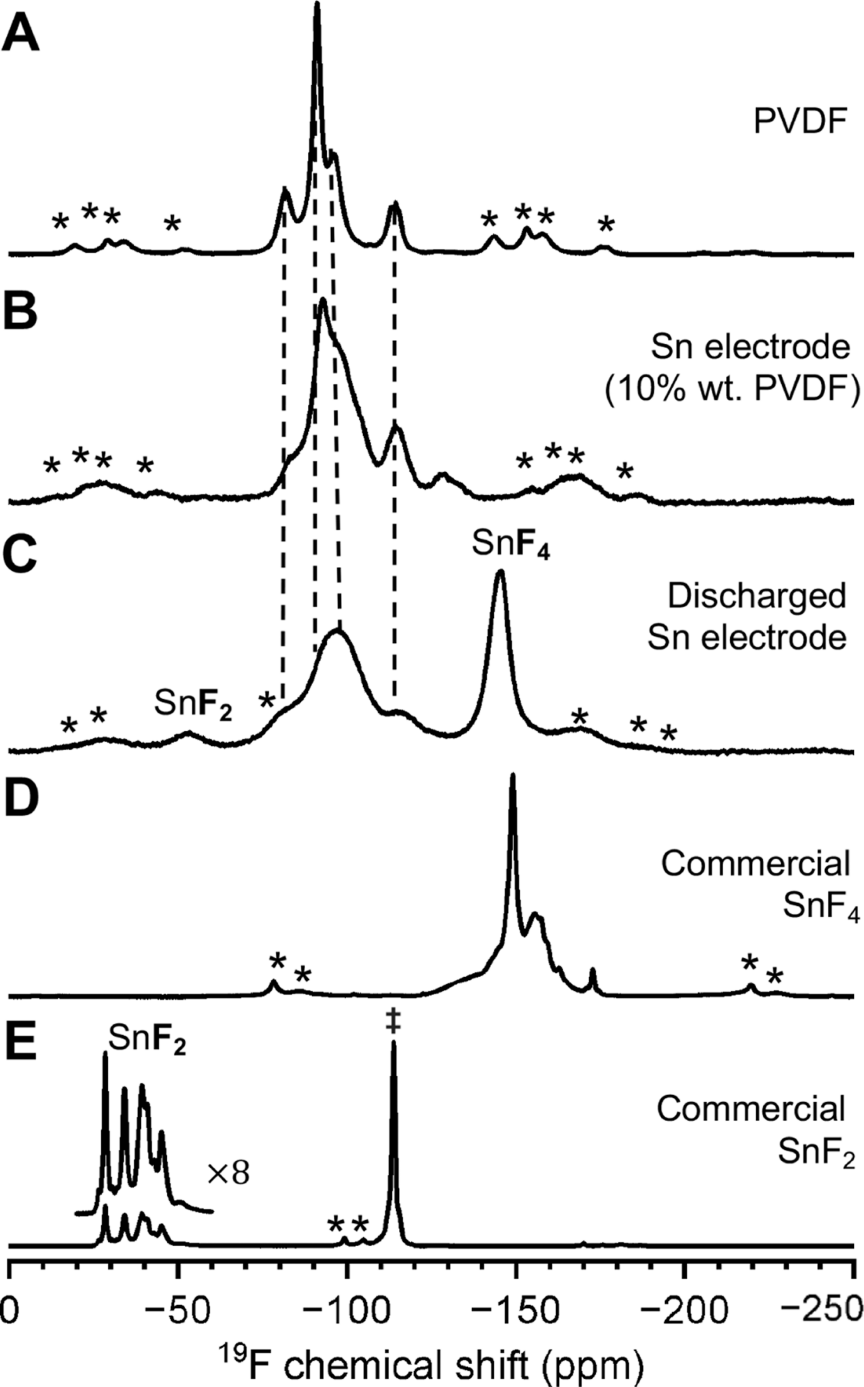
Solid-state ^19^F spin-echo MAS NMR spectra of (A) PVDF, (B) pristine Sn electrode, (C) discharged Sn electrode from the F-ion Sn-CF_*x*_ cell, (D) commercial SnF_4_, and (E) commercial SnF_2_. Spinning sidebands are labeled with asterisks. The SnF_4_-based impurity in panel (D) is marked with a dagger. All spectra were acquired at 14.1 T and 40 kHz MAS, except for SnF_4_, which was acquired at 38 kHz MAS.

The solid-state ^19^F NMR spectrum of pristine PVDF powder (Figure 6A) exhibits five ^19^F signals at −82, −91, −96, −113, and −115 ppm, revealing multiple characteristic environments associated with different local PVDF structures.^20^ The dominant ^19^F resonance at −91 ppm is assigned to amorphous PVDF domains. The ^19^F signals at −82 and −96 ppm are associated with crystalline PVDF environments, while those at −113 and −115 ppm are assigned to regio-irregular structures.^20^ The solid-state ^19^F NMR spectrum of the composite pristine Sn electrode (Figure 6B), which contains 10 wt % PVDF binder, exhibits similar ^19^F signals to PVDF. However, the ^19^F signals broadened significantly, a consequence of increased structural disorder due to the ball milling process that was used to prepare the electrode. A weak ^19^F signal was also observed at −128 ppm, likely associated with an impurity formed during the ball milling process.

After electrochemical discharge to 0 V, the solid-state ^19^F NMR spectrum of the discharged Sn electrode (Figure 6C) reveals a dominant, well-resolved ^19^F signal at −145 ppm. As shown below, this ^19^F signal is associated with SnF_4_. A weaker ^19^F signal centered at −52 ppm is also observed, which is likely that of SnF_2_. The discharged composite electrodes were rinsed with ethanol prior to performing solid-state NMR measurements to eliminate any mobile species from the residual electrolyte salt, Np_1_F. The solid-state ^19^F NMR spectra of the rinsed and unrinsed electrodes are nearly identical (Figure S2), except for the presence of the Np_1_F, which was successfully removed by ethanol rinsing.

The solid-state ^19^F NMR spectra of commercially purchased SnF_4_ and SnF_2_ fluorides were acquired to identify the fluorine environments in the discharged Sn electrode. The solid-state ^19^F NMR spectrum of SnF_4_ (Figure 6D) reveals an intense, well-resolved ^19^F signal at −149 ppm, a broad signal centered at −157 ppm, and multiple weaker ^19^F signals between −120 and −175 ppm. The SnF_4_ structure has two crystallographic fluorine sites corresponding to bridging and terminal fluorine atoms; thus, only two ^19^F NMR signals are expected. Notably, this solid-state ^19^F NMR spectrum is similar to the one shown in Dorn et al., who also reported multiple ^19^F sites in this spectral region.^21^ A solid-state 2D ^19^F{^19^F} dipolar-correlation NMR experiment (Figure S3) reveals that these ^19^F signals are all within closer molecular proximity and thus are within the same phase.^21^ Clearly, significant structural and chemical disorder is present within SnF_4_, which could be the result of different polymorphic forms or impurities incorporated during the synthesis or handling.

The solid-state ^19^F NMR spectrum of SnF_2_ (Figure 6E) shows well-resolved ^19^F signals at −29, −34, −39, and −45 ppm as well as a partially resolved ^19^F signal at −41 ppm, which are associated with SnF_2_. In addition, a prominent ^19^F signal is observed at −114 ppm, ascribed to SnF_4_-based impurities. The ^19^F signals in the region of −30 to −50 ppm are indeed SnF_2_, as shown by Dorn et al. using 2D solid-state ^19^F{^119^Sn} through-bond correlation NMR experiments. Bräuniger et al. and van Wüllen et al. also resolved SnF_2_^19^F environments, respectively, in the same ^19^F chemical shift region.^22,23^ The SnF_2_ structure has four crystallographic fluorine sites;^23^ the appearance of at least one additional ^19^F signal in this region indicates some degree of local disorder.

Interestingly, Dorn et al. characterized SnF_2_ from two different chemical suppliers: one exhibited the expected ^19^F signals from SnF_2_, and the other—which had a solid-state ^19^F NMR spectrum very similar to the one here (Figure 6E)—had a dominant ^19^F signal near −110 ppm, which was assigned to SnF_4_-based impurities due to the ^19^F chemical shift and ^119^Sn chemical shift anisotropies.^21^ Here, the ^19^F signal at −114 ppm is also suspected to be derived from SnF_4_-based impurities. A solid-state 2D ^19^F{^19^F} NMR dipolar-correlation experiment (Figure S4) reveals ^19^F signals of SnF_2_ and impurities within the same phase, in agreement with local disorder. Additionally, a solid-state ^119^Sn{^19^F} HETCOR NMR spectrum (Figure S6) reveals a correlation between the ^19^F signal at −114 ppm and the ^119^Sn signals centered at −820 ppm.

**Figure 7:**
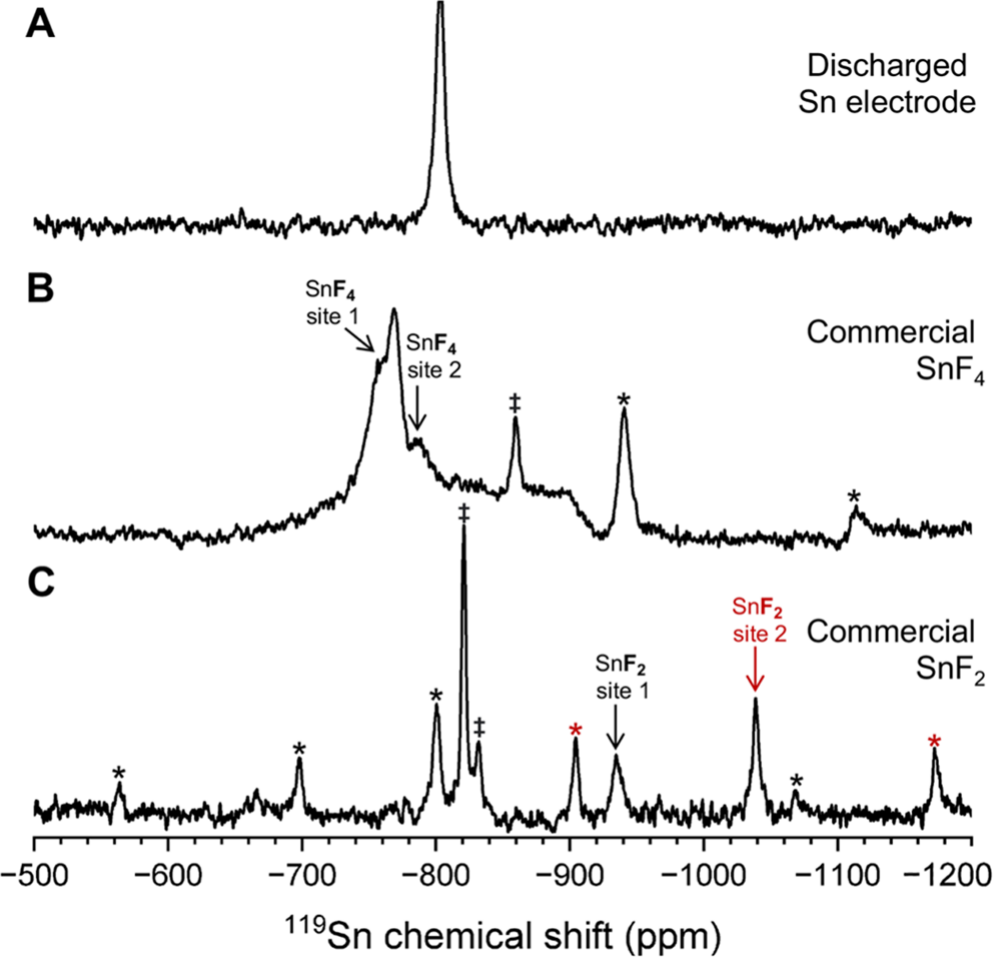
Solid-state ^119^Sn{^19^F} CP-MAS NMR of a (A) discharged Sn anode F-ion Sn-CF_*x*_ cell, (B) commercial SnF_4_, and (C) commercial SnF_2_. In panel (B), spinning sidebands associated with the ^119^Sn SnF_4_ signal at −767 ppm are labeled with black asterisks. In panel (C), spinning sidebands for SnF_2_ sites 1 and 2 are labeled with black and red asterisks, respectively. Impurities are marked with daggers. All spectra were acquired at 14.1 T and 40, 38, and 30 kHz MAS, respectively.

Solid-state ^119^Sn{^19^F} CP-MAS NMR measurements of the discharged Sn electrode were acquired to characterize ^119^Sn environments in subnanometer-scale proximity to ^19^F environments. The solid-state ^119^Sn{^19^F} CP-MAS NMR spectrum of the discharged Sn anode (Figure 7A) reveals a single ^119^Sn signal at −803 ppm. The ^119^Sn chemical shift is distinct compared to commercial SnF_4_ and SnF_2_ but notably is the same chemical shift region. The differences in the ^119^Sn chemical shift among these compounds are due to small changes in the local environment of Sn. A solid-state 2D ^19^F{^19^F} dipolar-correlation NMR experiment (Figure S5) also confirms that significant structural disorder is present within the fluorinated tin discharge products.

The solid-state ^119^Sn{^19^F} CP-MAS NMR spectrum of SnF_4_ (Figure 7B) reveals a dominant ^119^Sn signal at −767 ppm with a broad ^119^Sn shoulder at approximately −757 ppm, a weak, broad ^119^Sn signal at −785 ppm, and a sharp ^119^Sn signal at −858 ppm. The solid-state ^119^Sn{^19^F} CP-MAS spectrum is very similar to the one reported by Dorn et al. and Bräuniger et al. SnF_4_ contains two crystallographic tin sites; the ^119^Sn signals at −757 and −785 ppm are assigned to tin environments in coordination with terminal (site 1) and bridging (site 2) fluorine sites, respectively.^21^ Here, the ^119^Sn signals at −757 and −767 ppm may both be associated with terminal sites in slightly different local environments. The sharp ^119^Sn signal at −858 ppm is likely due to an impurity in a well-defined local environment.

The solid-state ^119^Sn{^19^F} CP-MAS NMR spectrum of commercial SnF_2_ (Figure 7C) reveals two isotropic ^119^Sn signals at −934 and −1039 ppm assigned to tin environments coordinated with three (site 1) or five fluorine (site 2) sites. SnF_2_ contains ^119^Sn in local coordination environments with either three or five fluorine atoms; thus, two isotropic ^119^Sn signals are expected.^21^ The intense, well-defined ^119^Sn signal at −820 ppm is ascribed to SnF_4_-based impurities, as discussed above, and is associated with the solid-state ^19^F spin-echo NMR spectrum signal at −114 ppm.

**Figure 8:**
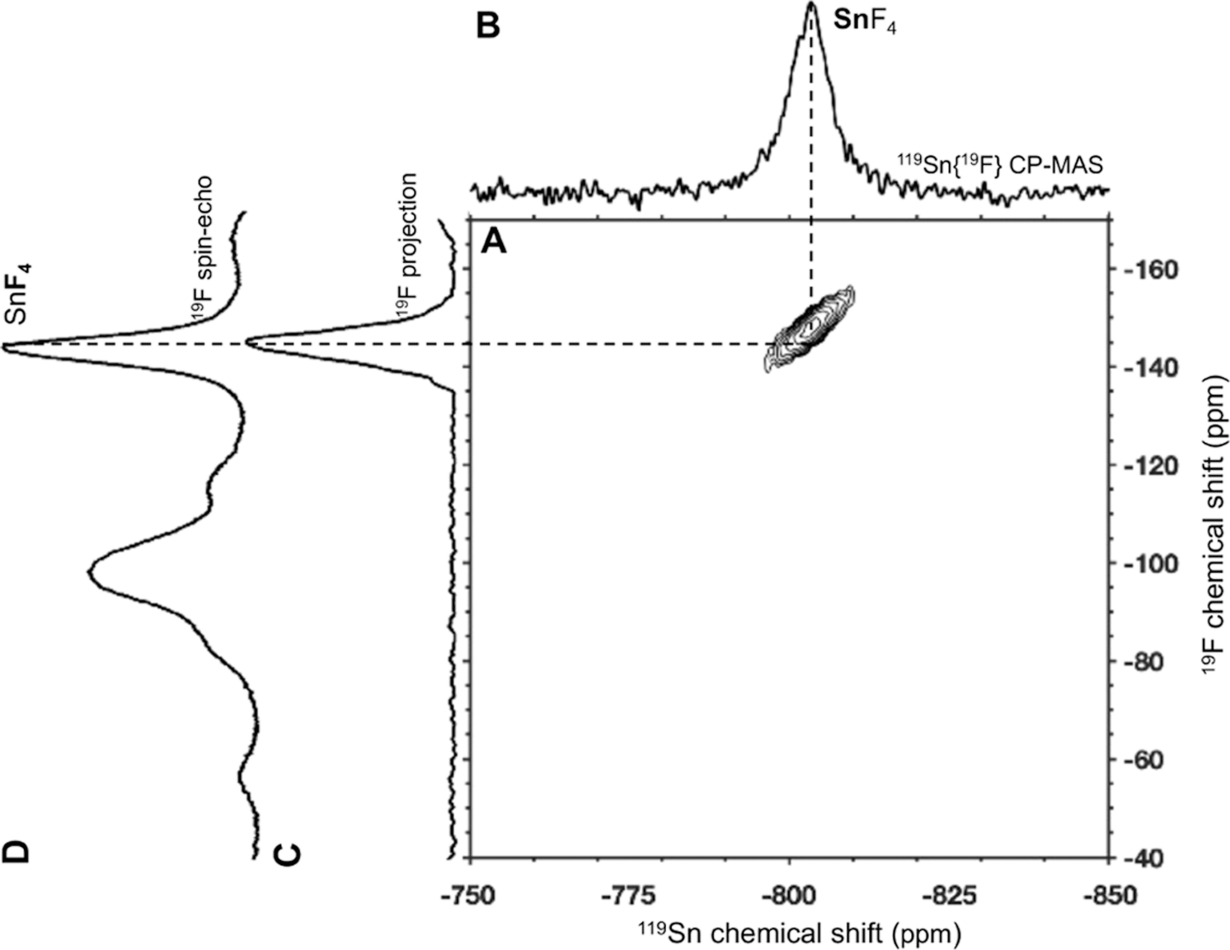
Solid-state NMR experiments performed on a discharged Sn electrode from a F-ion Sn-CF_*x*_ cell acquired at 40 kHz MAS and 14.1 T. (A) 2D ^119^Sn{^19^F} HETCOR spectrum, (B) separately acquired ^119^Sn{^19^F} CP-MAS spectrum, (C) ^19^F internal projection of the 2D HETCOR spectrum, and (D) separately acquired ^19^F spin-echo NMR spectrum.

Solid-state 2D dipolar mediated techniques were used to selectively probe interactions between ^19^F signals that are dipole–dipole coupled and, thus, in close molecular proximity to the ^119^Sn signals of the discharged Sn electrode. A solid-state ^119^Sn{^19^F} HETCOR NMR spectrum of the discharged Sn electrode (Figure 8) reveals a 2D correlated signal between the ^119^Sn at −803 ppm and ^19^F at −145 ppm, providing molecular-level evidence that SnF_4_ is electrochemically formed upon discharge. 2D correlations were not observed between any ^119^Sn signals and the ^19^F signal at −52 ppm associated with SnF_2_; this is likely the result of the lower concentration of the SnF_2_ species in the bulk Sn electrode.

**Figure 9:**
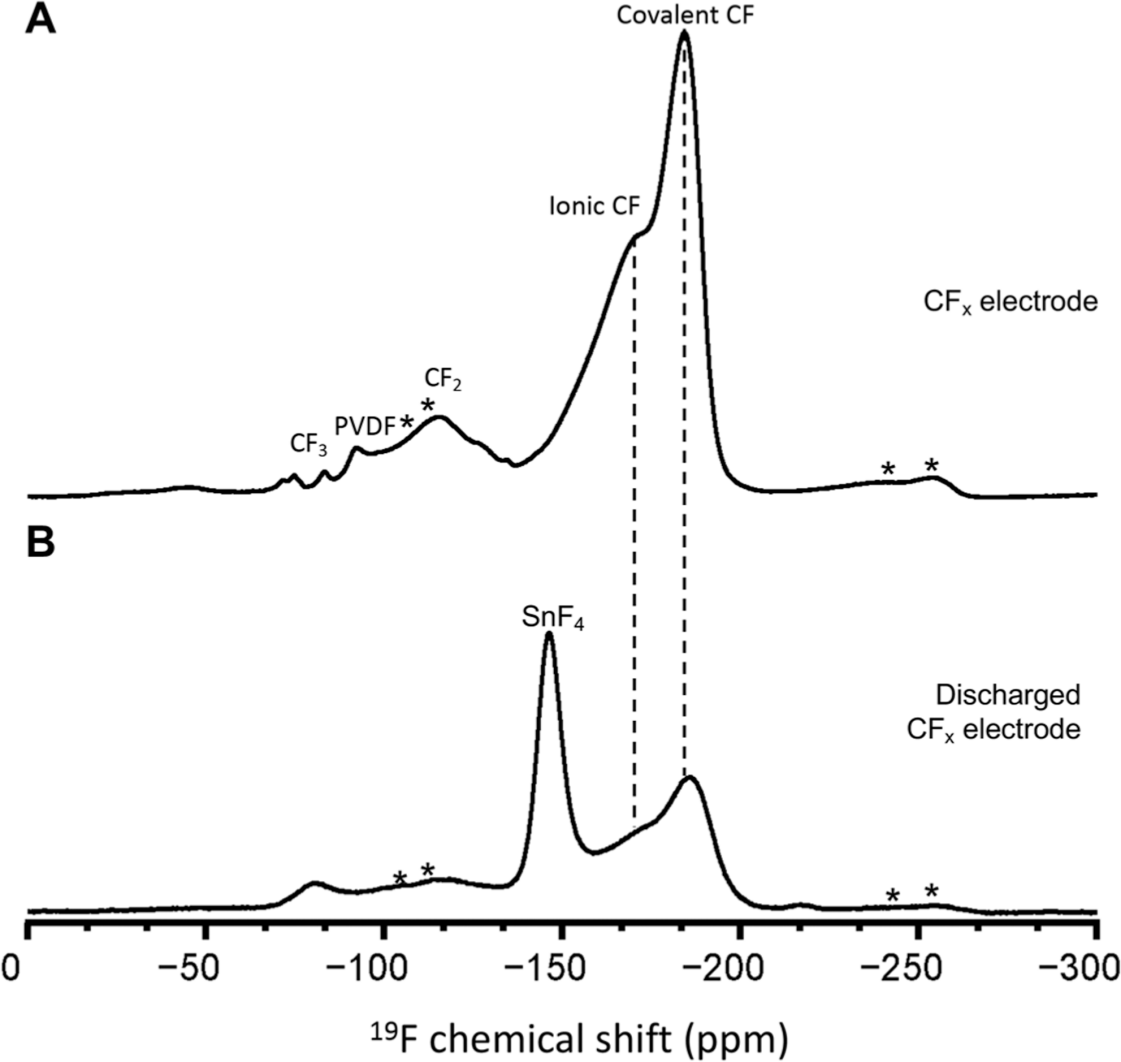
Solid-state ^19^F spin-echo MAS NMR spectra of (A) CF_*x*_ electrode and (B) discharged CF_*x*_ electrode from a F-ion Sn-CF_*x*_ cell. Spinning sidebands are labeled with an asterisk. All spectra were acquired at 14.1 T and 40 kHz MAS.

The solid-state ^19^F NMR spectrum of a pristine CF_*x*_ electrode (Figure 9A) reveals two intense ^19^F signals at −184 and −170 ppm attributed to covalent and semi-ionic CF bonds.^24^ Semi-ionic C–F bonds may also be described as covalent in nature but with a contribution from hyperconjugation.^25,26^ The C–F bond order is lowered within the CF_*x*_ structure due to hyperconjugation within the C–C bonds, resulting in a distortion from its covalent nature.^27^ This distortion changes the local fluorine electronic environment, altering the ^19^F NMR chemical shift. The broad ^19^F signal centered at −112 ppm and multiple well-resolved low-intensity signals between −70 and −90 ppm are associated with CF_2_ and CF_3_ sites. The ^19^F signal centered at −91 ppm corresponds to the amorphous domain of PVDF. As previously discussed, the PVDF binder exhibits multiple ^19^F signals depending on the local structure. The CF_*x*_ composite electrode consists of only 3 wt % PVDF; thus, the PVDF signals are weak in intensity.

Upon discharge, the ^19^F NMR spectrum of the discharged CF_*x*_ electrode (Figure 9B) reveals that ^19^F signals of CF covalent and semi-ionic fluorine environments decreased significantly with respect to the pristine CF_*x*_ electrode, as observed by the decrease in their relative ^19^F signal intensities. Critically, these results establish at the molecular level that CF_*x*_ is electrochemically defluorinated upon discharge. A well-resolved ^19^F signal at −145 ppm associated with SnF_4_ is observed, establishing that the mass transport of tin can occur from the Sn anode to the CF_*x*_ cathode. SnF_2_ was not observed in the discharged CF_*x*_ electrode, likely due to its low concentration in the bulk electrode sample. Sn transport was detected by XRF and solid-state NMR but not by XRD or Raman measurements. This apparent discrepancy may be due to the nature of the different measurement techniques and the amorphous nature of the electrochemically formed SnF_4_: XRD detects only reflections of crystalline components, while Raman scattering is limited to a specific region and depth resolution on the electrode surface.

To enable the relative ^19^F populations to be quantified, the solid-state ^19^F MAS NMR spectra of pristine CF_*x*_ and discharged CF_*x*_ and Sn electrodes were deconvoluted into individual ^19^F signals. The discharged Sn and CF_*x*_ electrodes were harvested from a cell that achieved a specific capacity of 314.9 mAh g^–1^, where the fraction of capacity extracted from the cell relative to the theoretical value of CF_*x*_ (865 mAh g^–1^, for *x* = 1) is 36.4%. The ^19^F molar populations of covalent and semi-ionic CF environments relative to the PVDF binder were 83 and 44% for the pristine and discharged CF_*x*_ electrodes, respectively, or a decrease of 39% (Figure 9). Note that the full theoretical specific capacity was not achieved by using these in-house Sn-CF_*x*_ cells, which were not experimentally optimized to extract maximum capacity. The ^19^F molar percentage decrease of 39% in the CF_*x*_ is in excellent agreement with the fraction of capacity extracted from the cell. Moreover, the ca. 2.6% discrepancy between the extracted capacity and the decrease in the ^19^F CF molar percentage in the CF_*x*_ electrode confirms that ∼3% of the discharge capacity was achieved by Sn^2+^ transport. The ^19^F molar population percentages of SnF_2_ and SnF_4_ present in the discharged Sn electrode (8.6 and 91.4%, respectively) were compared to the percentage of capacity extracted during the two plateaus observed during galvanostatic discharge (8.4 and 91.6%, respectively). The first and second discharge plateaus are therefore ascribed to the electrochemical formation of SnF_2_ and SnF_4_ from Sn metal, respectively, as they are in agreement with the quantified ^19^F molar populations.

**Figure 10:**
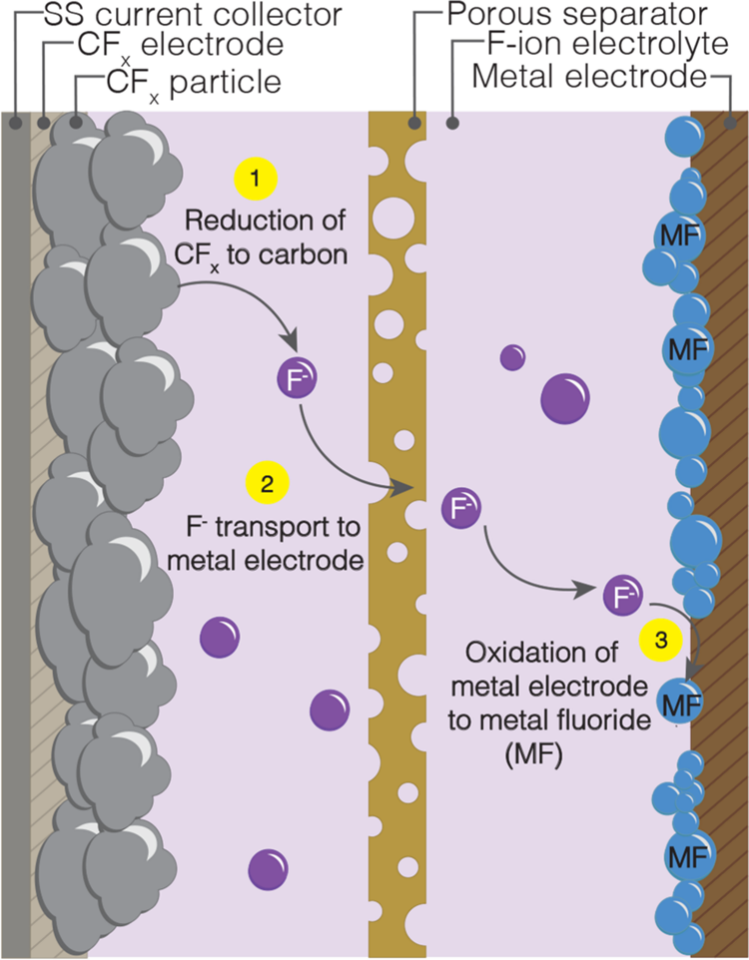
Schematic illustrating the electrochemical discharge of an F-ion metal-CF_*x*_ primary (nonrechargeable) cell.

The electrochemical discharge of an F-ion metal-CF_*x*_ cell is summarized schematically in Figure 10. The conversion mechanism of an F-ion metal anode-CF_*x*_ cell is the cathodic reduction of the CF_*x*_ electrode to form fluoride ions (F^–^) and carbon (C) (eq 1)
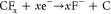
1the transport of fluoride ions through the electrolyte, and the anodic oxidation of the metal electrode (M) to form metal fluorides (MF) with the discharge products remaining at the anode (eq 2).
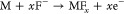
2The overall electrochemical reaction is shown in eq 3.
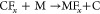
3

## Conclusions

In this work, we demonstrate the electrochemical defluorination of CF_*x*_ cathodes using a room-temperature F-ion conducting electrolyte when paired with either a Pb or Sn anode. The primary F-ion Pb-CF_*x*_ and Sn-CF_*x*_ cells achieved capacities of 700 and 400 mAh g^–1^, respectively, without optimization. XRD measurements show that the metal fluorides PbF_2_ or SnF_2_ form on the Pb or Sn anodes, respectively. Solid-state ^19^F and ^119^Sn{^19^F} NMR measurements of an F-ion Sn-CF_*x*_ discharged Sn electrode and electrode components revealed the presence of both SnF_2_ and SnF_4_, where SnF_4_ is the main discharge product. Solid-state 2D ^19^F{^19^F} dipolar-correlation NMR experiments of the discharged Sn electrode revealed the amorphous nature of the electrochemically formed SnF_4_, confirming why it was not observed by XRD. The presence of nano-ordered regions, with some amorphous in nature, is also validated by Raman scattering and surface morphology changes of the electrodes upon discharge.

Metal fluorides were discovered on the discharged CF_*x*_ cathode of both the F-ion Pb-CF_*x*_ and Sn-CF_*x*_ cells, as evidenced by XRD, XRF, and solid-state NMR. ICP, XRF, and solid-state NMR quantified a ca. 5.4 and 3.1% contribution to the discharge capacity due to Pb^2+^ and Sn^2+^ transport and subsequent reaction. Thus, a >40% reduction in metal fluoride transport to the CF_*x*_ electrode was achieved when using Sn, compared to the Pb. Solid-state ^19^F NMR measurements of a pristine and discharged CF_*x*_ electrode from a Sn-CF_*x*_ cell establish unambiguously that the C–F bonds are broken upon discharge. The ^19^F molar percentage of CF bonds consumed (39%) was compared to the cell capacity extracted related to the theoretic capacity of CF_*x*_ (36.4%), confirming the electrochemical defluorination of CF_*x*_ as well as a minor contribution due to Sn cation transport. Overall, we have demonstrated a new electrochemical discharge mechanism relative to conventional Li-CF_*x*_ cells that reduces polarization loss and raises the possibility of improvement to a higher practical discharge voltage. Future work will address the lithium metal counter electrode reactivity to enable an F-ion-based Li-CF_*x*_ cell.

## Supporting Information Available

Structural parameters obtained from the Rietveld refinements, XRD patterns of electrode components and electrodes, solid-state ^19^F NMR spectra of electrolyte salt and electrodes, solid-state 2D ^19^F{^19^F} and^119^Sn{^19^F} MAS experiments, supporting text, and calculations (PDF)
