# Supplementary material for: Development of Fluoride-Ion Primary Batteries: The Electrochemical Defluorination of CFx
Source: J Phys Chem C Nanomater Interfaces. 2024 Aug 15;128(34):14195–205. doi: 10.1021/acs.jpcc.4c03412 (PMC11372825; doi:10.1021/acs.jpcc.4c03412)
Supplement: Supplementary file 1 — jp4c03412_si_001.pdf [file jp4c03412_si_001.pdf]

Supporting Information

Development of Fluoride-ion Primary Batteries: The Electrochemical Defluorination of CF_x

Loleth E. Robinson[a], Jonah Wang[a], Harrison Asare[a,b,c], Jessica L. Andrews[d], Balram Tripathi[f], Ram Katiyar[f], Brent C. Melot[d,e], Robert J. Messinger[a,c*], Simon C. Jones[g,*], William C. West[h,*]

[a]*Department of Chemical Engineering, The City College of New York, CUNY, New York, NY 10031, USA*

[b]*Department of Chemistry and Biochemistry, The City College of New York, CUNY, New York, NY 10031, USA*

[c]*Ph.D. Program in Chemistry, The Graduate Center of The City University of New York, New York, NY 10016, USA*

[d]*Department of Chemistry, University of Southern California, Los Angeles, CA 90089, USA*

[e]*Department of Chemical Engineering and Materials Science, University of Southern California, Los Angeles, CA 90089, USA*

[f]*Department of Physics and Institute for Functional Nanomaterials, University of Puerto Rico, San Juan, 00925-2537, Puerto Rico*

[g]*Flion Energy Inc., Pasadena, CA 91107, USA*

[h]*Jet Propulsion Laboratory, California Institute of Technology, Pasadena, CA 91109, USA*

[*]E-mail: rmessinger@ccny.cuny.edu, simon.jones@flionenergy.com, william.c.west@jpl.nasa.gov

Table S1. Structural parameters obtained from the Rietveld refinements of a discharged CF_x electrode from a F-ion Pb-CF_x cell.

**Parameter**	**PbF_2**
**Space group**	*Fm*-3*m* (#225)
a	5.9449(2) Å
b	5.9449(2) Å
c	5.9449(2) Å
α	90°
β	90°
γ	90°
**Pb1**	(0, 0, 0)
**F1**	(¼, ¼, ¼)
**Weight %**	100%
**Mole %**	100%
**R_{wp}**	3.407

Table S2. Structural parameters obtained from the Rietveld refinements of a discharged Pb electrode from a F-ion Pb-CF_x cell.

**Parameter**	**PbF_2**	**Pb**	**PbO**	**PbF_2**
**Space group**	*Pnma* (#62)	*Fm*-3m (#225)	*Pbcm* (#57)	*Fm*-3m (#225)
a	6.4480(2) Å	4.95161(7) Å	5.8926(3) Å	5.9423(4) Å
b	3.9015(1) Å	4.95161(7) Å	5.495(1) Å	5.9423(4) Å
c	7.6549(2) Å	4.95161(7) Å	4.750(1) Å	5.9423(4) Å
α	90°	90°	90°	90°
β	90°	90°	90°	90°
γ	90°	90°	90°	90°
**Pb1**	(0.2568(7), ¼, 0.1059(2))	(0, 0, 0)	(0.239(1), 0.035(2), ¼)	(0, 0, 0)
**F1**	(0.867(2), ¼, 0.080(2))	–	–	(¼, ¼, ¼)
**F2**	(0.439(2), ¼, 0.842(2))	–	–	–
**O1**	–	–	(–0.132, 0.082, ¼)	–
**Weight %**	82.25%	7.64%	8.64%	1.47%
**Mole %**	80.44%	8.84%	9.28%	1.44%
R_{wp}	4.589	–	–	–

Table S3. Structural parameters obtained from the Rietveld refinements of a pristine Sn electrode.

Parameter	Sn
**Space group**	*I*4$_1$/*amd* (#141)
a	5.83255(2) Å
b	5.83255(2) Å
c	3.18232(1) Å
α	90°
β	90°
γ	90°
**Sn1**	(0, 0, 0)
**Weight %**	100%
**Mole %**	100%
R_{wp}	6.247

Table S4. Structural parameters obtained from the Rietveld refinements of a discharged Sn electrode from a F-ion Sn-CF_x cell.

Parameter	Sn	SnF_2
**Space group**	$I4_1/amd$ (#141)	$C12/c1$ (#15)
a	5.8314(9) Å	13.355(3) Å
b	5.8314(9) Å	4.9089(9) Å
c	3.1814(7) Å	13.788(3) Å
α	90°	90°
β	90°	109.12(2)°
γ	90°	90°
**Sn1**	(0, 0, 0)	(0.3716(6), 0.474(1), 0.0566(7))
**Sn2**	–	(0.1116(4), 0.483(1), 0.1872(5))
**F1**	–	(0.234(3), 0.180(8), 0.302(4))
**F2**	–	(0.407(2), 0.235(7), 0.183(3))
**F3**	–	(0.225(3), 0.356(7), 0.045(3))
**F4**	–	(0.483(4), 0.341(8), 0.417(4))
**Weight %**	27.86%	72.14%
**Mole %**	33.76%	66.24%
R_{wp}	6.143	–

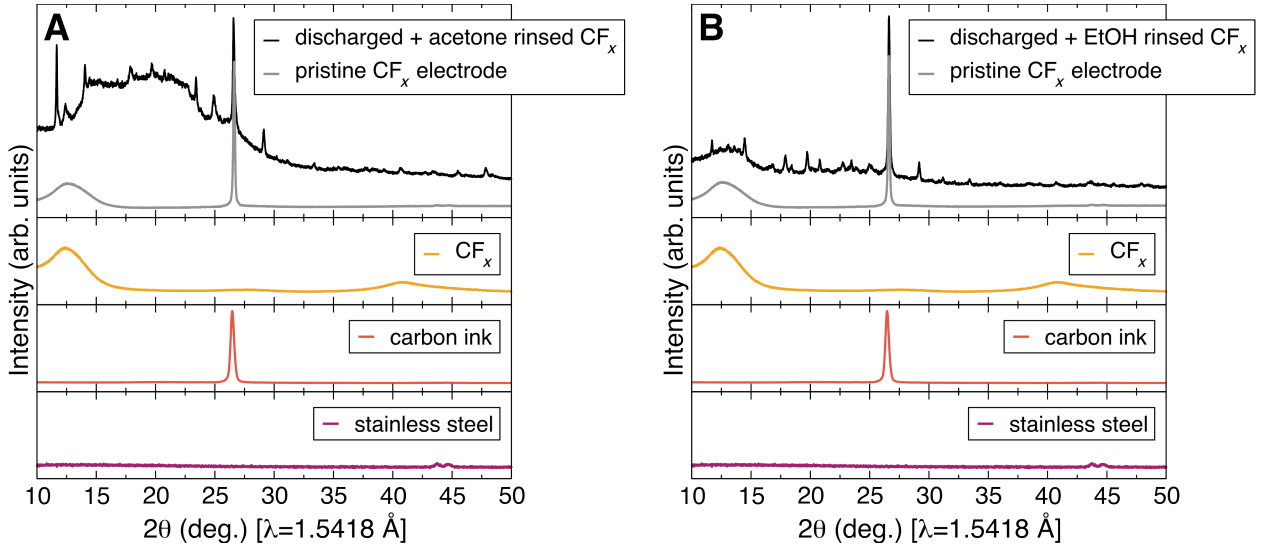

Figure S1. XRD pattern of a discharged CF_x electrode rinsed with (A) acetone (black) or (B) ethanol (black). XRD patterns of a pristine CF_x electrode (gray), CF_x powder (yellow), carbon ink (orange) and stainless-steel foil (purple) are shown below each discharged CF_x electrode. Both discharged CF_x electrodes were harvested from a F-ion Sn-CF_x cell.

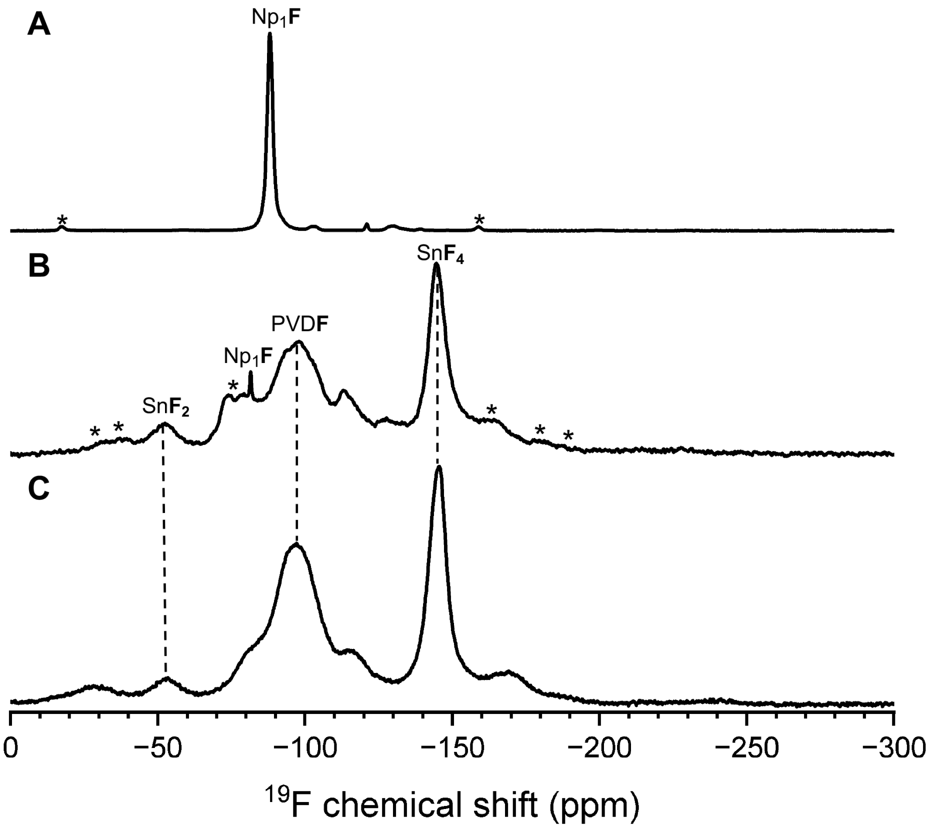

Figure S2. Solid-state ^{19}F spin-echo MAS NMR spectra of (A) Np_1F, (B) unrinsed discharged Sn electrode and (C) discharged Sn electrode from a F-ion Sn-CF_x cell rinsed with ethanol. Spinning sidebands are labeled with asterisks. The ^{19}F Np_1F signal from residual electrolyte is labeled in (B). All spectra were acquired at 40 kHz MAS and 14.1 T.

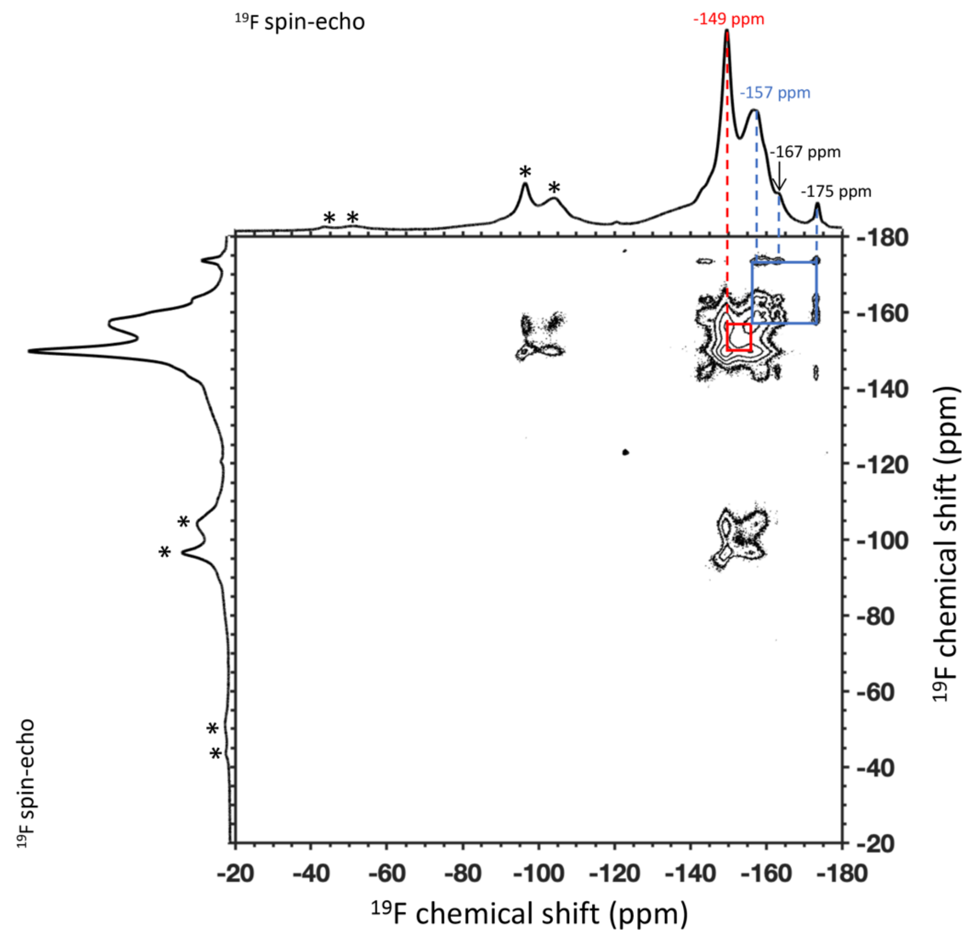

Figure S3. Solid-state 2D $^{19}F\{^{19}F\}$ fp-RFDR dipolar correlation NMR spectrum of SnF_4 acquired a mixing time of 1.6 ms under conditions of 30 kHz MAS and 14.1 T. Spinning sidebands are labeled with asterisks. Separately acquired solid-state ^{19}F spin-echo NMR spectra are shown along the horizontal and vertical axes for reference. Solid lines indicate correlated 2D signal intensity between the two dominant ^{19}F signals associated with SnF_4 environments and multiple weak ^{19}F environments, establishing their mutual dipole-dipole interactions and hence sub-nanometer-scale proximities.

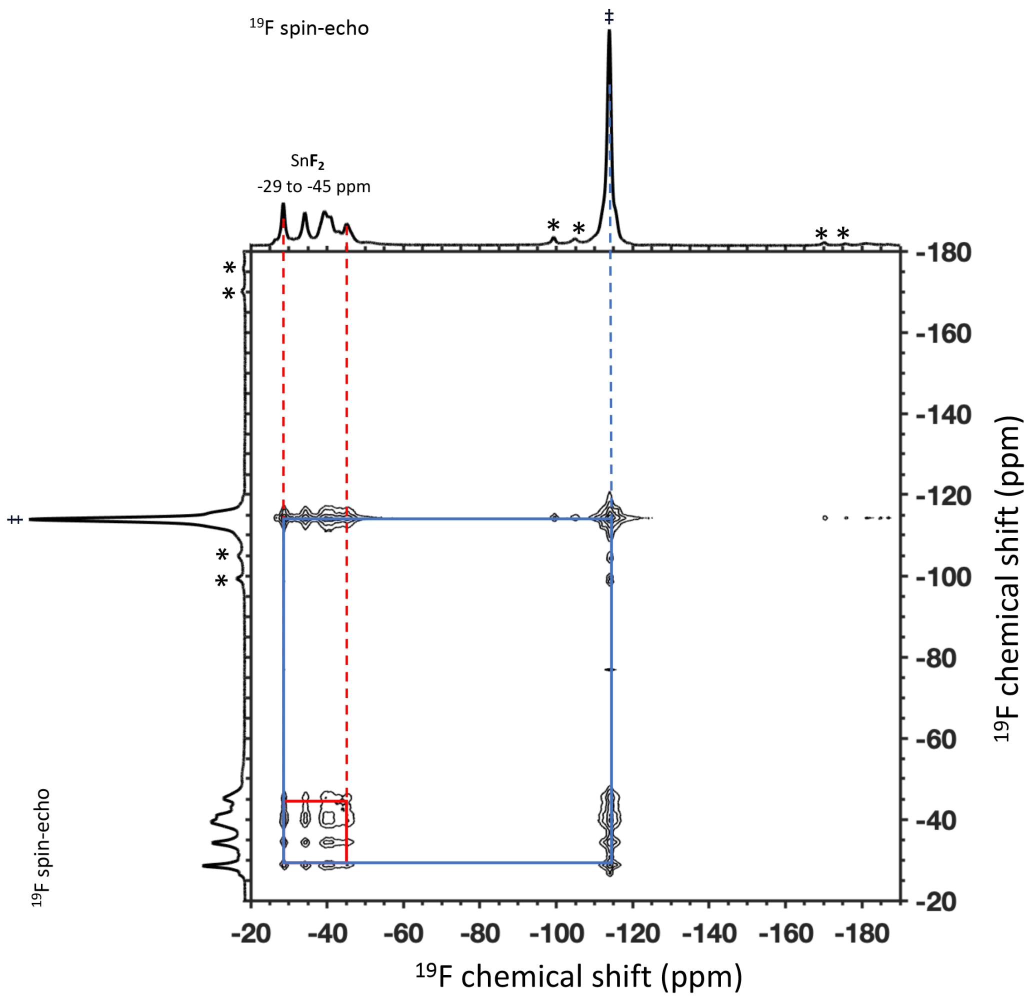

Figure S4. Solid-state 2D $^{19}F\{^{19}F\}$ fp-RFDR dipolar correlation NMR spectrum of SnF_2 acquired a mixing time of 1.6 ms under conditions of 40 kHz MAS and 14.1 T. Spinning sidebands are labeled with asterisks. Separately acquired solid-state ^{19}F spin-echo NMR spectra are shown along the horizontal and vertical axes for reference. The SnF_4-based impurity is marked with a dagger. Solid lines indicate correlated 2D ^{19}F signals associated with SnF_2 environments, as well as between SnF_2 and the impurity, establishing their mutual dipole-dipole interactions and hence sub-nanometer-scale proximities.

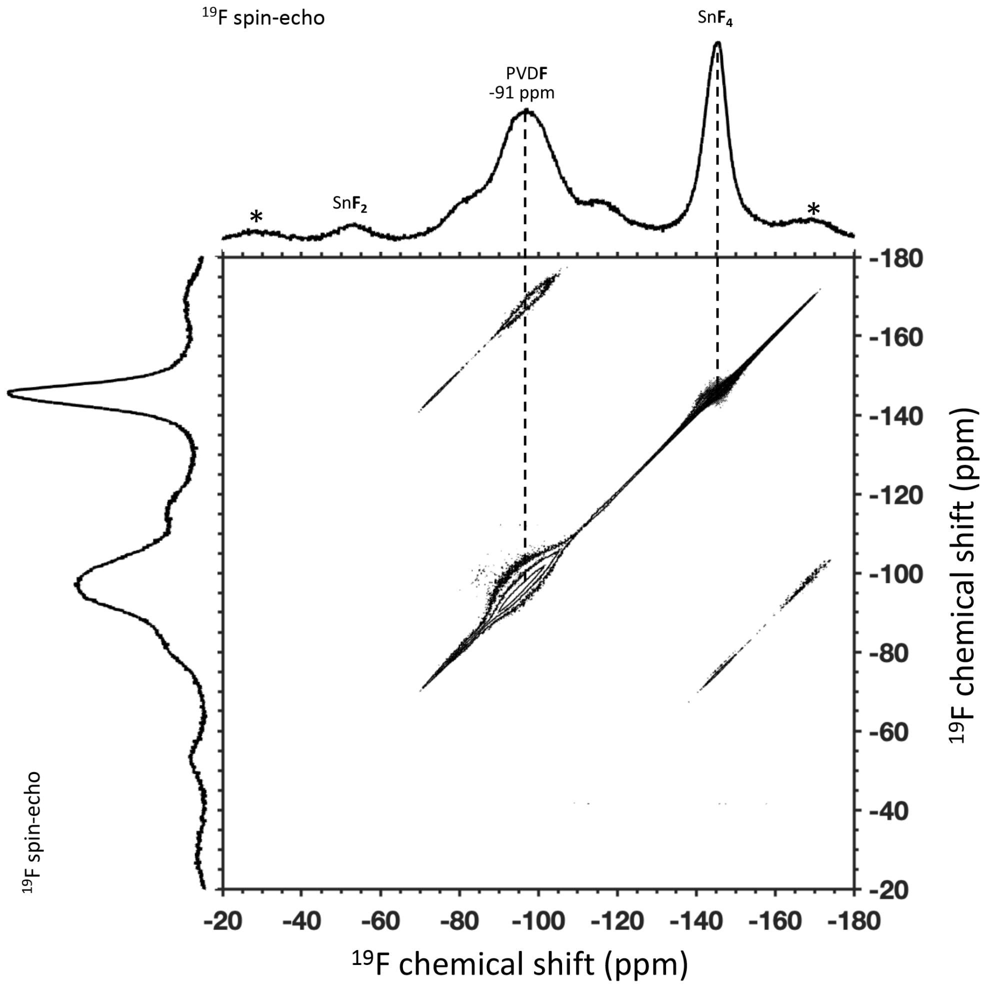

Figure S5. Solid-state 2D $^{19}F\{^{19}F\}$ fp-RFDR dipolar correlation NMR spectrum of a discharged Sn electrode from a F-ion Sn-CF_x cell acquired with a mixing time of 1.6 ms under conditions of 40 kHz MAS and 14.1 T. Spinning sidebands are labeled with asterisks. Separately acquired solid-state ^{19}F spin-echo NMR spectra are shown along the horizontal and vertical axes for reference. 2D correlations were not observed within the ^{19}F SnF_4 environments, confirming the amorphous nature of the discharge product. Also, 2D correlations were not observed between ^{19}F PVDF and SnF_4 environments, consistent with their local phase segregation.

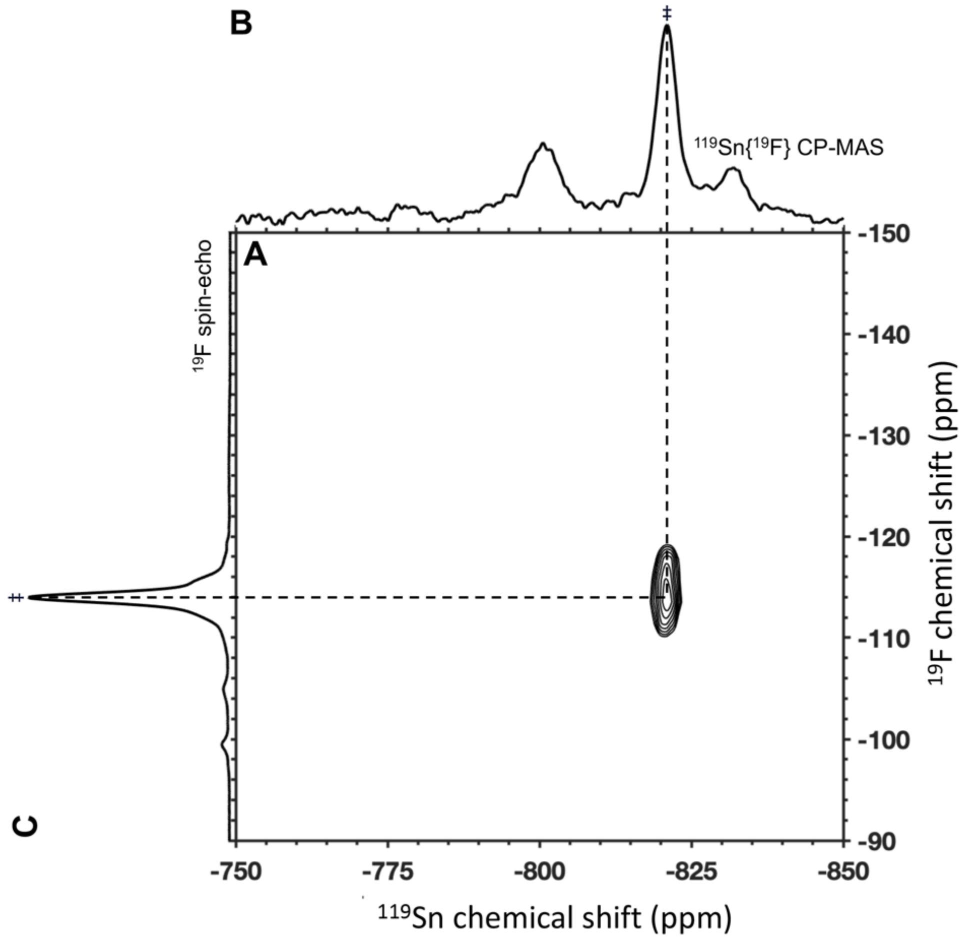

Figure S6. Solid-state NMR experiments performed on SnF_2 acquired at 40 kHz MAS and 14.1 T. (A) 2D ^{119}Sn{^{19}F} HETCOR NMR spectrum, (B) separately acquired ^{119}Sn{^{19}F} CP-MAS NMR spectrum, and (C) separately acquired ^{19}F spin-echo NMR spectrum.

Supporting Text & Calculations

Text S1: Calculation of the specific capacity.

All metal-CF_x cells were constructed with the metal anode specific capacity far in excess of the CF_x cathode specific capacity; thus, they are all cathode-limited in capacity by design. Specific capacities are reported per mass of CF_x (865 mAh g^{-1}, based on CF_x cathode when $x \approx 1$). The composite CF_x electrodes consisted of 92 wt.% CF_x powder ($x \approx 1$), 5 wt.% carbon black, and 3 wt.% polyvinylidene fluoride. The specific capacity in mAh g^{-1} was calculated as follows:

$$Specific\ capacity\ (mAh\ g^{-1}) = \frac{Discharge\ capacity\ (mAh)}{grams\ of\ CF_x}$$

where

$$grams\ of\ CF_x = composite\ electrode\ mass\ (g) \times CF_x\ mass\ loading\ (\%)$$

Text S2: Estimation of the quantity of metal fluorides present on the CF_x electrodes.

The quantity of metal fluoride present on discharged CF_x electrodes harvested from different F-ion Sn-CF_x cells was 3.8%, 3.0%, and 2.6% as determined by ICP, XRF and ^{19}F solid-state NMR measurements, respectively. We report a 3.1% estimate of tin fluoride on the CF_x electrode as an average from the results of the three different characterization methods. Estimates obtained from each method are detailed below.

ICP: ICP acquired on a discharged tin electrode harvested from a F-ion Sn-CF_x cell reveals that the tin fluoride discharge products contain 4.77 mg of Sn and 1.52 mg of fluorine. The estimated mass of fluorine removed from the CF_x electrode based on the specific capacity achieved from the harvested electrode was 1.58 mg. Therefore,

$$\frac{experimental\ mass, fluorine}{theoretical\ mass, fluorine} = \frac{1.52\ mg}{1.58\ mg} \times 100 = 96.2\%$$

The theoretical mass of fluorine was calculated using Faraday's law:

$$mass\ (mg) = \frac{Q}{F} \times \frac{M}{z}$$

where Q is the capacity, F is Faraday's constant, M is the molar mass and z is the number of electrons transferred.

Thus, ca. 96.2% of the discharge product was located in the tin anode and ca. 3.8% on the CF_x cathode, as quantified by ICP.

XRF: XRF acquired on a discharged CF_x electrode harvested from a F-ion Sn-CF_x reveals that the electrode contained 2.5 wt.% of Sn. The estimated capacity extracted from Sn metal migration to the CF_x electrode was calculated using Faraday's law of electrolysis and equates to 0.135 mAh. The total capacity obtained from the cell was 4.46 mAh. Therefore,

$$\frac{Capacity\ extracted, Sn\ metal\ migration}{Total\ capacity} = \frac{0.135\ mAh}{4.46\ mAh} \times 100 = 3.0\%$$

Thus, ca. 3.0% of the capacity was due to Sn transport to the CF_x cathode as quantified by XRF.

Solid-state NMR: Deconvolutions of the solid-state ^{19}F MAS NMR spectra of a pristine and discharged CF_x harvested from a F-ion Sn-CF_x cell reveal a 39% decrease in the ^{19}F molar populations of covalent and semi-ionic CF environments in CF_x. The discharged Sn and CF_x electrodes were harvested from a cell that achieved a specific capacity of 314.9 mAh g^{-1}, where the fraction of capacity extracted from the cell relative to the theoretical value of CF_x (865 mAh g^{-1}, for x=1) was 36.4%. Therefore,

$$\begin{aligned} &Fraction\ of\ capacity\ extracted\ from\ cell\ relative\ to\ CF_x \\ &\quad - decrease\ in\ 19F\ molar\ populations\ of\ CF\ environments\ in\ CF_x \\ &\quad = 39\% - 36.4\% = 2.6\% \end{aligned}$$

Thus, ca. 2.6% of the discharge capacity was due to Sn transport as quantified by solid-state ^{19}F NMR measurements.
